# Multimerization Increases Tumor Enrichment of Peptide–Photosensitizer Conjugates

**DOI:** 10.3390/molecules24040817

**Published:** 2019-02-25

**Authors:** Jisi Zhao, Shuang Li, Yingying Jin, Jessica Yijia Wang, Wenjing Li, Wenjie Wu, Zhangyong Hong

**Affiliations:** 1 College of Material Science and Chemical Engineering, Tianjin University of Science and Technology, Tianjin 300457, China; 13920398911@163.com; 2 State Key Laboratory of Medicinal Chemical Biology, College of Life Sciences, Nankai University, Tianjin 300071, China; lishuang5258@163.com (S.L.); 18222912650@163.com (Y.J.); l800450@163.com (W.L.); 3 Tianjin Sirui International School, Sisui Road, Hexi District, Tianjin 300222, China; jessica.Wang@students.tiseagles.com

**Keywords:** photodynamic therapy, pyropheophorbide-a, RGD peptide, integrin, photosensitizer

## Abstract

Photodynamic therapy (PDT) is an established therapeutic modality for the management of cancers. Conjugation with tumor-specific small molecule ligands (e.g., short peptides or peptidomimetics) could increase the tumor targeting of PDT agents, which is very important for improving the outcome of PDT. However, compared with antibody molecules, small molecule ligands have a much weaker affinity to their receptors, which means that their tumor enrichment is not always ideal. In this work, we synthesized multimeric RGD ligand-coupled conjugates of pyropheophorbide-a (Pyro) to increase the affinity through multivalent and cluster effects to improve the tumor enrichment of the conjugates. Thus, the dimeric and trimeric RGD peptide-coupled Pyro conjugates and the monomeric one for comparison were efficiently synthesized via a convergent strategy. A short polyethylene glycol spacer was introduced between two RGD motifs to increase the distance required for multivalence. A subsequent binding affinity assay verified the improvement of the binding towards integrin α_v_β_3_ receptors after the increase in the valence, with an approximately 20-fold improvement in the binding affinity of the trimeric conjugate compared with that of the monomeric conjugate. In vivo experiments performed in tumor-bearing mice also confirmed a significant increase in the distribution of the conjugates in the tumor site via multimerization, in which the trimeric conjugate had the best tumor enrichment compared with the other two conjugates. These results indicated that the multivalence interaction can obviously increase the tumor enrichment of RGD peptide-conjugated Pyro photosensitizers, and the prepared trimeric conjugate can be used as a novel antitumor photodynamic agent with high tumor enrichment.

## 1. Introduction

Photodynamic therapy (PDT) is an established therapeutic modality for the treatment of a variety of premalignant and malignant diseases [1,2]. It utilizes a photosensitizer activated by light to create irreversible photodamage to tumor tissues [3,4]. Compared with traditional therapeutic methods, such as chemotherapy, radiotherapy, and surgery, PDT has the advantages of minimal invasiveness and extremely low systemic toxicity [5]. Furthermore, PDT agents can act as fluorescent probes for in vivo cancer diagnoses and presentations, naturally combining fluorescence imaging and cancer therapy in a “see and treat” manner [6,7]. Recently, it was also found that PDT can induce strong antitumor immune responses and thus contribute to the reduction of the residual tumor burden following surgical resection of tumors [8,9]. For these reasons, PDT has emerged as an important therapeutic option for the management of cancers.

The enrichment of photosensitizers at tumor sites is very important for improving the treatment effect of PDT [10]. It can increase the photosensitizer concentration in tumor sites, which is beneficial for improving the photodynamic killing ability and thus is very important for the treatment of large or deep-seated tumors [11]. What is more, considerable damage occurs to the surrounding normal tissues if the photosensitizers that are used lack adequate tumor selectivity, because it is always difficult to precisely distinguish tumors from normal tissues by using precise light illumination because of the complex tumor infiltration in normal tissues [12]. Because of this nonselectivity, the fluorescence detection capability is also detrimentally influenced. Based on this, much research has been focused on developing strategies to enhance the enrichment of photosensitizers at tumors sites.

The conjugation between photosensitizers and tumor homing ligands, such as monoclonal antibodies, epidermal growth factors, or peptide ligands, is a promising strategy for optimizing the tumor targeting of PDT agents [13,14]. For example, Yamagaci et al. synthesized tumor-targeting photosensitizers by conjugating photosensitizer IR700 with antibody molecules, and the synthesized conjugates showed excellent tumor selectivity and enrichment towards receptor-positive tumors [15]. The improved tumor enrichment using this strategy considerably improved the outcome of PDT, as demonstrated in the tumor treatment in a mouse model, and currently, they are undergoing advanced clinical trials, which represents a very promising and powerful method of cancer therapy. To overcome the difficulties in using large proteins and antibodies as targeting vehicles, conjugating with various tumor-specific small molecule ligands (e.g., short peptides or peptidomimetics) would provide another promising strategy because of the unique advantages of small molecule ligands [16]. For example, unlike the obtained antibody conjugates, which are always complex mixtures because of random site conjugation [17], small molecule ligands may easily afford conjugates with a single structure. Second, conjugates with small molecule ligands have a relatively short circulation half-life, which is beneficial for reducing the phototoxic side effects to the skin and eye that are otherwise due to the long-term presence of photosensitizers in the body. Furthermore, the stability and preparation costs of conjugates with small molecule ligands are also important issues for application. Thus, the development of photosensitizers conjugated with small molecule ligands has been extensively studied [18,19,20,21].

However, compared with antibody molecules, small molecule ligands have a much weaker affinity to their receptors, which makes their tumor enrichment not always ideal. In our former work, we coupled a very promising photosensitizer, pyropheophorbide-a (Pyro), which has ideal photosensitizer properties, with a small molecule tumor homing ligand—cyclo(-RGDfK-)—to prepare a tumor-targeted conjugate [22]. The cyclic peptide cyclo(-RGDfK-), which contains a conformationally restrained RGD sequence, has been often used for targeting tumor imaging and/or therapeutic agents as a high affinity ligand for the α_v_β_3_ integrin receptor [23,24,25,26,27,28,29,30,31]. This conjugate showed improved tumor enrichment compared to free Pyro, thus greatly improving the PDT outcome against tumors in a mouse model and destroying implanted tumors with only one or two treatments of PDT. However, the tumor enrichment capability of this conjugate is still not very good because the cyclo(-RGDfK-) ligand only has a moderate affinity to the α_v_β_3_ integrin receptor compared with antibody molecules. Improving the affinity of small molecule ligands is very critical for improving the tumor enrichment of small molecule ligand-based conjugates and is thus very important for improving their clinical application potential.

To improve the affinity of RGD ligand-coupled Pyro conjugates for α_v_β_3_ integrin targeting, we decided to synthesize multimeric conjugates with a polyvalent RGD sequence that can bind simultaneously to several integrins. Multimeric ligands have a higher receptor binding affinity and better tumor retention because the polyvalence results in enhanced binding and steric stabilization [32,33]. Multivalent interactions are thought to be a very practical strategy for amplifying weak ligand–receptor interactions that are more biologically relevant, thus affording more effective ligands with better targeting capability. For example, Chen [34] and Ryppa [35] et al. constructed conjugates of paclitaxel with multivalent RGD peptides, and the conjugates showed enhanced selective killing of cancer cells, excellent integrin specific accumulation and very good tumor/background contrast in vivo. However, until now, not much attention has been paid to preparing the conjugates of photosensitizers with multimeric ligands. To this end, we hope to adopt a multivalence strategy to improve the tumor enrichment capability of the RGD-coupled Pyro photosensitizer to enhance its clinical application potential.

## 2. Results and Discussion

### 2.1. Molecular Design

**Figure 1:**
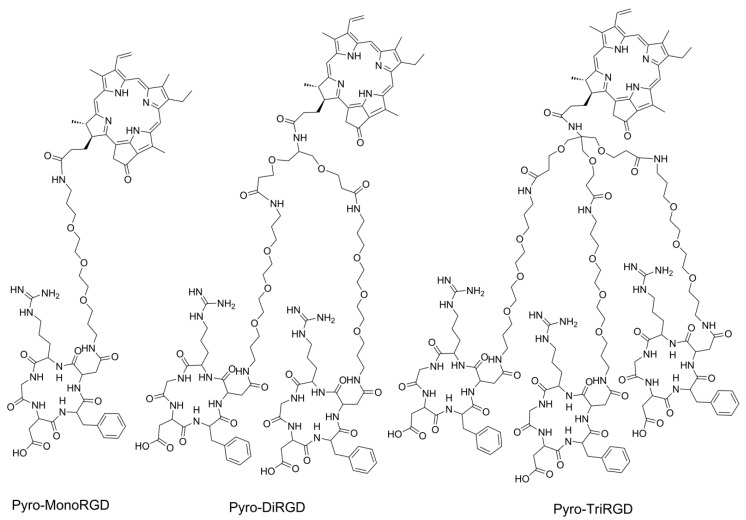
Structures of the Pyro-MonoRGD, Pyro-DiRGD, and Pyro-TriRGD conjugates.

Pyro is a very promising photosensitizer with ideal photoactivity properties, including a high extinction coefficient (3.79 × 10^4^ L mol^−1^ cm^−1^) and a high singlet oxygen quantum yield (over 50%) [36]. However, Pyro has very limited tumor localization ability. Solving the tumor enrichment problem is very important for its clinical application. Here, to further increase the affinity of RGD peptide-coupled Pyro conjugates to the receptor α_v_β_3_ integrin, we synthesized multimeric RGD-coupled Pyro conjugates to increase the affinity via multivalent and cluster effects, thereby improving the uptake selectivity and tumor enrichment of the conjugates by tumor tissues. Thus, in this study, we synthesized the dimeric and trimeric RGD peptide-coupled Pyro conjugates and the monomeric one to compare and optimize the effect of multivalence (Figure 1). Since an exchange of valine in the cyclic pentapeptide cyclo(-RGDfV-) for another amino acid has no significant influence on the activity or selectivity [37], we used cyclo(-RGDfD-), which offers a free carboxylic acid group for peptide coupling reactions, as the ligand for the convenience for molecular and functional modifications. The distance between two RGD motifs in multimeric RGD peptides is very important for multivalence [38]. It must be long and flexible enough to achieve simultaneous integrin α_v_β_3_ binding. Thus, we introduced a polyethylene glycol moiety of similar length as reported in the literature [39] as the spacer. The use of a polyethylene glycol spacer is desirable because of its high hydrophilicity, which is helpful to increase the solubility of peptide conjugates and for extending the ligand out for receptor binding and for reducing the influence of the Pyro macrocycle on the receptor affinity of the RGD ligand.

To synthesize these conjugates, we adopted a convergent strategy in which we used a solid-phase strategy based on Fmoc chemistry to prepare the cyclic RGD peptide molecule with a carboxylic group that is free for conjugation and with all other groups protected. Then, we used solution reactions to assemble the building blocks of Pyro, multivalent/monomeric linkers, and the side chain-protected RGD peptide ligand together. This convergent synthesis strategy affords these conjugates in a very efficient manner.

### 2.2. Solid-Phase Synthesis of Side Chain-Protected Cyclic RGD Pentapeptide 1 and 2

**Scheme 1:**
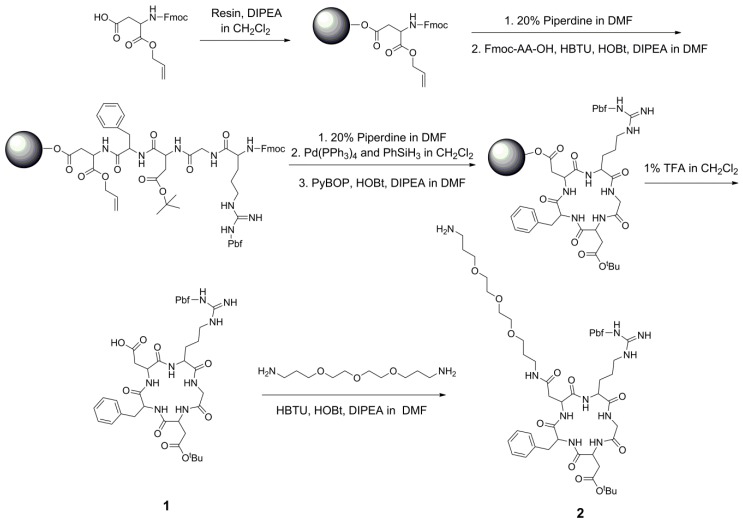
Synthetic route for cyclic RGD pentapeptide **1** and amino-modified cyclic RGD peptapeptide **2**.

The synthesis started from the preparation of the cyclic RGD peptide ligands (Scheme 1). Here, we used the strategy of Fmoc solid-phase peptide synthesis to prepare the side chain-protected cyclic RGD peptide ligand: cyclic pentapeptide cyclo(-Arg[Pbf]GlyAsp[tBu]-D-Phe-Asp-) **1**. It conveniently contains a free carboxylic acid group for the following derivatization. For its synthesis, Fmoc-Asp-OAll was first attached to the 2-chlorotrityl chloride resin, followed by coupling with D-Phe, Asp, Gly, and Arg. After removing the Fmoc group using piperidine and the OAll group by Pd(PPh_3_)_4_, the peptide was cyclized on the resin. Then, the peptide was cleaved from the resin suing 1% TFA in CH_2_Cl_2_ with all side chain groups protected, and it was purified using a silica gel column to produce the protected pentapeptide cyclo(-Arg[Pbf]GlyAsp[tBu]-D-Phe-Asp-) **1** in a yield of 42%. Then, to add a hydrophilic polyethylene glycol (PEG) linker as a spacer between Pyro and the RGD ligand, the pentapeptide **1** was reacted with 4,7,10-Trioxa-1,13-tridecanediamine to produce the amino group-modified RGD peptide **2** in an 81% yield. It is worth mentioning that both RGD peptides **1** and **2** could be easily purified with a conventional silica gel column. Through this on-resin cyclization and the following amino group derivatization in-solution reaction, the side chain-protected RGD ligand was obtained very easily with one free carboxylic acid or one amino group for the following derivatization, offering a very simple method for synthesis of RGD-coupled conjugates.

### 2.3. Synthesis of Mono-RGD Conjugate Pyro-MonoRGD

**Scheme 2:**
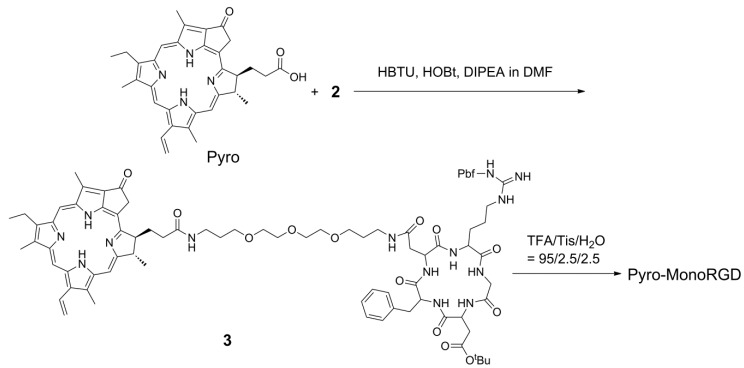
Synthetic route for the mono-RGD conjugate, Pyro-MonoRGD.

The Pyro molecule has one free carboxylic acid group, which can be used for coupling with amino-modified RGD ligand **2** via the typical condensation reaction between a carboxylic group and an amino group (Scheme 2). The condensation between RGD peptide **2** and Pyro afforded compound **3** in an 80% yield. After removing the protective group on the RGD moiety with 95% TFA, the monomeric RGD peptide-coupled Pyro conjugate (Pyro-MonoRGD) was obtained as a solid in a 71% yield after HPLC purification. The product was characterized by mass spectrometry and HPLC (Supplementary Materials, Figures S1 and S4). Mass spectrometry showed that it has a molecular weight of 1309.6694, which is consistent with its theoretical mass of 1309.6734. HPLC showed that the product was of a very good purity.

### 2.4. Synthesis of the Dimeric-RGD Conjugate, Pyro-DiRGD

**Scheme 3:**
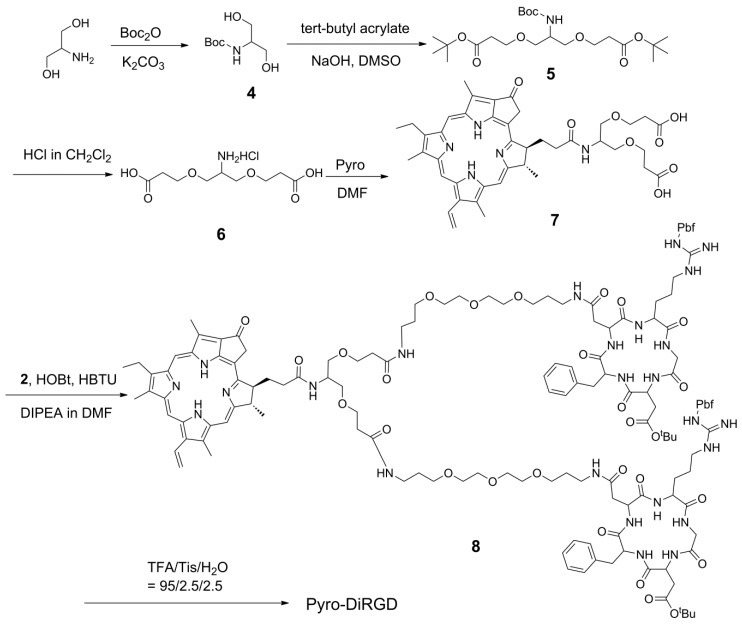
Synthetic route for the di-RGD conjugate, Pyro-DiRGD.

For the synthesis of the dimeric RGD-coupled Pyro conjugate Pyro-DiRGD (Scheme 3), a proper dimeric linker was first prepared. Here, 2-amino-1,3-propanediol was first tested to directly condense with tert-butyl acrylate in the presence of NaOH as a base; however, we could not obtain the desired product. We could only obtain the addition product in which the amino group reacted with tert-butyl acrylate. The amino group seems to have a higher reaction rate than the hydroxyl group to react with tert-butyl acrylate in 2-amino-1,3-propanediol. Thus, 2-amino-1,3-propanediol was first protected with the tert-butyloxycarbonyl (Boc) group to afford derivative **4** in a 62% yield and then condensed with tert-butyl acrylate to afford **5** in a 58% yield. After deprotection of the Boc and tert-butyl groups, the obtained compound **6** was first assembled with the preformed activated ester of Pyro at room temperature to afford the Pyro coupled dimeric linker **7** in a yield of 92% and then subsequently coupled with amino-modified RGD peptide **2** to produce the side chain-protected dimeric RGD-coupled Pyro conjugate **8** in a yield of 72%. It is worth mentioning that both Pyro-coupled dimeric linker **7** and dimeric RGD-coupled Pyro conjugate **8** can be easily purified with a conventional silica gel column, making this method very convenient for amplifying the synthesis. The desired dimeric RGD conjugate Pyro-DiRGD was then obtained in a 67% yield after simple deprotection and HPLC purification. The product was characterized via mass spectrometry and HPLC, and the relative data are presented in the Supplementary Materials (Figures S1 and S5).

### 2.5. Synthesis of the Trimeric-RGD Conjugate, Pyro-TriRGD

**Scheme 4:**
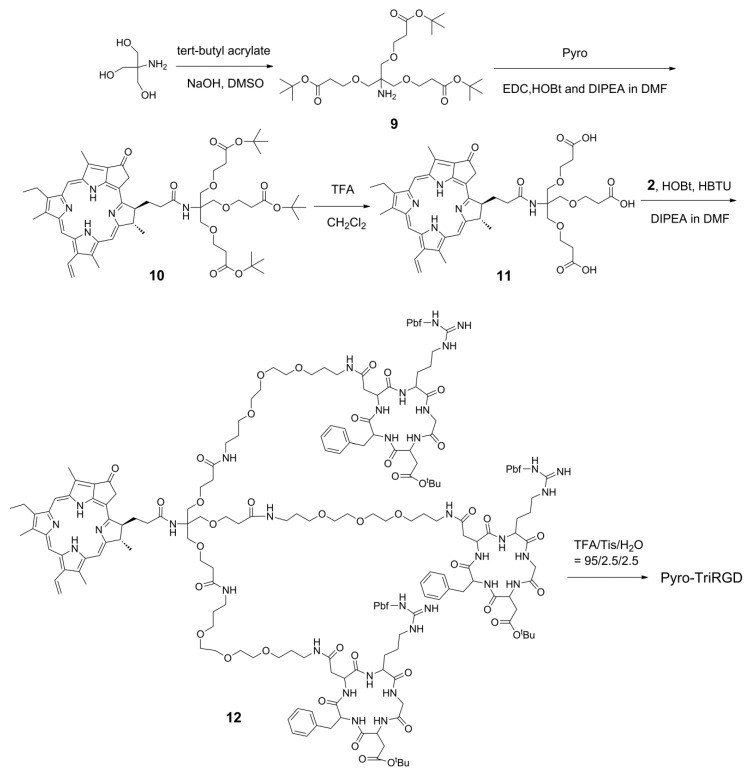
Synthetic route for the Pyro-TriRGD conjugate.

The synthesis of the trimeric RGD-coupled Pyro conjugate, Pyro-TriRGD, began with the preparation of the trimeric linker with the functional amino and carboxylic groups for coupling (Scheme 4). Here, tris(hydroxymethyl)aminomethane (Tris base) was condensed with tert-butyl acrylate in the presence of NaOH without protection of the amino group to afford the trimeric linker **9** in a 38% yield. In contrast to 2-amino-1,3-propanediol, the free amino group in the Tris base seemed to be not able to compete with the hydroxyl group for the condensation with tert-butyl acrylate, possible due to the strong steric hindrance. Thus, there is no necessity to protect the free amino group in the Tris base for this condensation. Trimeric linker **9** was assembled with the preformed activated ester of Pyro to obtain the Pyro-coupled trimer Linker **10** in a yield of 92%. Deprotection with TFA afforded **11**, which was then subsequently coupled with amino group-modified RGD peptide **2** to produce the side chain-protected trimeric RGD-coupled Pyro conjugate **12** in **a** 62% yield. Here, both Pyro-coupled trimer Linker **10** and side chain-protected RGD-coupled with the Pyro conjugate **12** could also be easily purified with a silica gel column. Then, the desired product, Pyro-TriRGD, was obtained in a 70% yield after simple deprotection and HPLC purification. The product was characterized by mass spectrometry and HPLC, and the relative data are presented in the Supplementary Materials (Figures S1 and S6).

### 2.6. Photophysical and Photochemical Properties

The photophysical and photochemical properties of Pyro-MonoRGD, Pyro-DiRGD, Pyro-TriRGD, and free Pyro were measured and re summarized in Table 1. All these conjugates exhibited very similar UV–Vis spectra and fluorescence spectra compared with free Pyro with an intense and sharp Q-band at 667–668 nm, a fluorescence excitation band at approximately 668 nm and an emission band at 672–673 nm. The singlet oxygen quantum yields (Φ_Δ_) of these conjugates were in the range of 0.47 to 0.49, which were also comparable with that of free Pyro (Φ_Δ_ = 0.52). These data show that the RGD peptide conjugation did not obviously affect the photophysical or photochemical properties of Pyro.

### 2.7. Receptor Binding Assay

**Figure 2:**
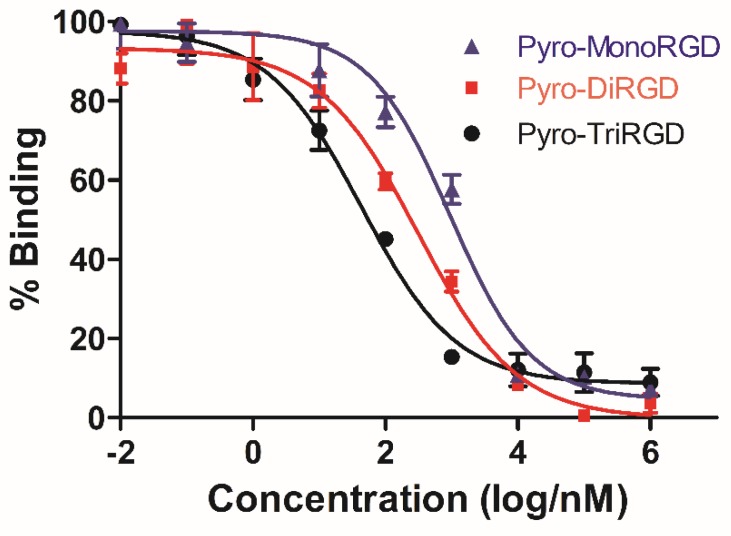
Comparison of the inhibitory action of Pyro-MonoRGD, Pyro-DiRGD, and Pyro-TriRGD on binding of integrin α_v_β_3_ and vitronectin. Data are expressed as the means ± SD (n = 3).

To evaluate the influence of multivalent conjugation on the binding capability of the RGD conjugate, the ability of the three conjugates—Pyro-MonoRGD, Pyro-DiRGD, and Pyro-TriRGD—to inhibit the binding of biotinylated vitronectin to human isolated α_v_β_3_ integrin receptors in vitro was determined by a competitive binding assay (Figure 2 and Table 2). Here, compared with the monovalent RGD conjugate of Pyro-MonoRGD (IC_50_ = 950.7 ± 39.6 nM), the binding of the divalent conjugated Pyro-DiRGD (IC_50_ = 313.2 ± 23.1 nM) to integrin α_v_β_3_ increased. An even better improvement was observed for the trivalent conjugate, Pyro-TriRGD (IC_50_ = 43.8 ± 8.2 nM), which binds integrin α_v_β_3_ 20-fold better than the monovalent counterpart, Pyro-MonoRGD, and 7-fold better than the divalent Pyro-DiRGD. These values suggest that the multivalent interactions play important roles in increasing the binding affinity of conjugates. It can enhance the binding strength to a greater extent than the sum of the affinity of the involved ligands, which is somewhat proportional to the number of conjugated ligands.

### 2.8. In Vivo Distribution Analysis

**Figure 3:**
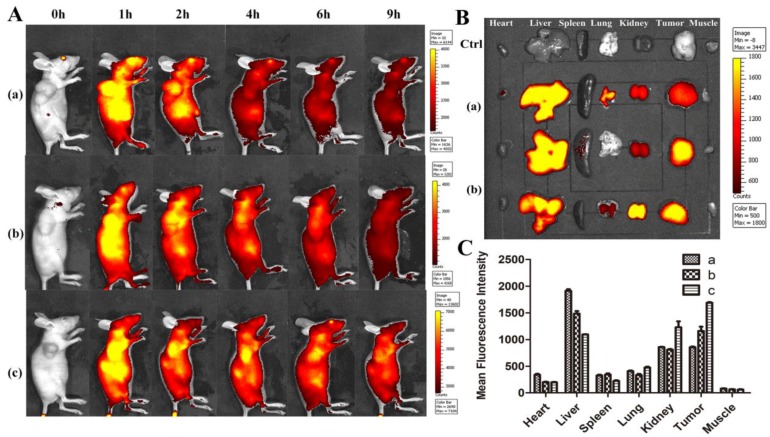
Drug accumulation and retention studies in U87-MG tumor-bearing nude mice. (**A**) In vivo fluorescence imaging of Pyro-MonoRGD (a), Pyro-DiRGD (b), and Pyro-TriRGD (c) obtained at 0, 1, 2, 4, 6, and 9 h after injection of the corresponding conjugates. Fluorescent signals of the different conjugates were imaged (**B**) and quantitative detected (**C**) in the organs and tumors 2 h post-administration (a, b, and c represent Pyro-MonoRGD, Pyro-DiRGD, and Pyro-TriRGD, respectively). The results are shown as averages ±SD of triplicates.

To examine the tumor enrichment of these conjugates in vivo, we prepared a xenograft tumor model by subcutaneously inoculating mice with U87-MG tumor cells (1.0 × 10^6^ cells per mouse) on the axilla. U87-MG cells have high expression levels of the α_v_β_3_ integrin receptor. When the tumors grew to approximately 200 mm^3^ in volume, the conjugates (50 nmol per mouse) were intravenously injected into the mice via the tail vein. The fluorescence signal and intensity distribution were continuously monitored using an in vivo fluorescence imaging system (IVIS Lumina II, Xenogen, Alameda, CA, USA). As shown in Figure 3A, these conjugates were rapidly distributed throughout the body, and an intense fluorescence signal was primarily located in the organs of the liver, kidney, and tumor one hour postinjection. At two hours postinjection, the fluorescence signals in the liver obviously decreased. For the Pyro-MonoRGD conjugate, at two hours postinjection, the tumor-to-background ratios increased, and accumulation in the tumor could be observed; however, the signals for the fluorescence in the liver were still very strong. Afterwards, the signals both in the liver and tumor disappeared quickly, and almost disappeared at four hours postinjection. For the Pyro-DiRGD conjugate, the tumor distribution was significantly different. At two hours postinjection, the fluorescence signal of Pyro-DiRGD in the tumor site was already stronger than the one in the liver, and this strong fluorescence intensity in the tumor site lasted until 4 h postinjection. However, relatively weak fluorescence signals in the tumor site could be detected. For the Pyro-TriRGD conjugate, after two hours postinjection, it was mainly concentrated in the tumor site, and its signal became even stronger at four hours postinjection, at which time the signals were almost completely located in the tumor area and only negligible signals could be found in other tissues. More importantly, the signal in the liver region sequentially weakened from Pyro-MonoRGD, Pyro-DiRGD, to Pyro-TriRGD. The increased water hydrophilicity of the divalent and trivalent conjugates may contribute to the reduction in the liver accumulation.

To further verify the tumor targeting ability of these conjugates, the mice were euthanized 2 h postinjection, and their organs were collected for imaging. As shown in Figure 3B (heart, liver, spleen, lungs, and tumor from left to right), when the valence of the conjugates increased, the fluorescence signal in the tumor region increased. Pyro-TriRGD exhibited the strongest fluorescence intensity in the tumor. The fluorescence intensity in these organs was also calculated and is shown in Figure 3C. Pyro-TriRGD exhibited the highest fluorescence intensity at the tumor site and the lowest intensity at the liver compared with the other two conjugates.

These initial data provide strong evidence for high-tumor targeting of multivalent RGD conjugates, especially the trimeric one. The trivalent conjugate, Pyro-TriRGD, exhibited much improved tumor enrichment ability with excellent tumor accumulation at 2–6 h postinjection, and it may have good potential for tumor targeting PDT. Additionally, the accumulation of the conjugates in the liver significantly reduced because of the multimeric conjugation, probably due to the increased water hydrophilicity of the divalent and trivalent conjugates.

## 3. Experimental

### 3.1. Instruments and Materials

Fmoc-Asp-OAll, Fmoc-Dphe-OH, Fmoc-Asp(O^t^Bu)-OH, Fmoc-Gly-OH, Fmoc-Arg(Pbf)-OH, *N*-hydroxybenzotriazole (HOBt), and 2-(1*H*-benzotriazol-1-YL)-1.1.3,3-tetramethyluronium hexafluorophosphate (HBTU) were purchased from GL Biochem Ltd. (Shanghai, China). Phenylsilane, triisopropylsilane (Tis), tetratriphenylphosphine palladium (Pd(PPh_3_)_4_), benzotriazol-1-yl-oxytripyrrolidinophosphonium hexafluorophosphate (PyBOP), diisopropylethylamine (DIPEA), trifluoroacetic acid (TFA), and *N*,*N*-dimethylformamide (DMF) were purchased from Heowns (Tianjin, China). ^1^H and ^13^C NMR spectra were recorded in CDCl_3_ or in D_2_O solutions on a Bruker AV400 or AV300 spectrometer (Bruker Bioscience, Billerica, MA, USA.) The Cary 5000 spectrophotometer (Varian Co., Palo Alto, CA, USA) was used for UV–Vis spectra. Fluorescence spectra were recorded using a Hitachi model F-4500 FL spectrophotometer (Tokyo, Japan). Mass data were obtained using a Varian 7.0 T FTMS. High-performance liquid chromatography (HPLC) (Shimadzu, Japan) was used for the analysis and purification of the conjugates. In vivo imaging analysis was performed using an IVIS Lumina imaging system (IVIS Lumina II, Xenogen, Alameda, CA, USA). Human glioblastoma U87-MG cells were purchased from a typical culture preservation commission cell bank of the Chinese Academy of Sciences (Shanghai, China) and were grown in DMEM culture medium, which contained L-glutamine, 10% fetal bovine serum (FBS), and 1% Pen/Strep (10 000 U penicillin, 10 mg streptomycin). BALB/c nude mice (male and female, 7−8 weeks old) were provided by Beijing Vital River Laboratory Animal Technology Co., Ltd. Animal studies were conducted in accordance with animal ethic guidelines and ethic approval (Number 20180002) by ethic committee on animals of Nankai University (Tianjin, China) which was abtained prior the study.

### 3.2. Solid-Phase Synthesis of Side Chain-Protected Cyclic RGD Pentapeptide Cyclo(-Arg[Pbf]-Gly-Asp[tBu]-D-Phe-Asp-)

The syntheses were manually performed in a sealed tube using a solid-phase strategy. 2-chlorotrityl chloride resins (8.0 g) were swollen in dichloromethane for 1 h prior to synthesis. Fmoc-Asp-OAll (949 mg, 2.4 mmol, 0.3 mmol/g loading) and DIPEA (2.0 mL, 5.0 mmol) were dissolved in 30 mL of dichloromethane and added to the resin. The mixture was agitated for 5 h. Afterwards, a sealing agent (CH_2_Cl_2_:MeOH:DIPEA = 17:1:2, 20 mL) was used to seal the unreacted chlorine group for 30 min. The Fmoc protecting group was removed by treatment with 20% piperidine in DMF (20 mL) for 30 min. Coupling reactions of the other four amino acids (Fmoc-D-Phe-OH, Fmoc-Asp(O^t^Bu)-OH, Fmoc-Gly-OH, and Fmoc-Arg(Pbf)-OH) were performed using 2 equiv of a Fmoc-protected derivative and activated in situ with HBTU (2.0 equiv), HOBt (1.5 equiv), and DIPEA (5 equiv) in DMF for 12 h. Excess reagents were washed five times with DMF for each coupling step.

After the final Fmoc deprotection, the resin was treated with PhSiH_3_ (24 equiv) and Pd(PPh_3_)_4_ (0.3 equivalents) in dichloromethane for 1 h under N_2_ protection for selective alpha-carboxyl deprotection of the allyl groups. Cyclization between α-NH_2_ of Arg and α-CO_2_H of Asp was conducted overnight in DMF using PyBOP (2 equiv)/HOBt (1.5 equiv)/DIPEA (5 equiv). The resin was washed with DMF and dichloromethane before it was dried under vacuum to afford the resin-bound cyclic RGD pentapeptide.

The peptide was cleaved from the resin by treating the dried resin with 5 mL of 1% TFA at room temperature 5 times, and excess TFA was neutralized with DIPEA. The mixture was concentrated under reduced pressure, and the solid was washed with dry diethyl ether (1.5 mL) three times. Then, the mixture was purified by column chromatography (silica gel, CH_2_Cl_2_-MeOH, 10:1) to afford pure peptapeptide **1** (57 mg, 42%). HRMS-ESI: *m*/*z* = 899.3956, calcd for C_42_H_59_N_8_O_12_S *m*/*z* = 899.3973 [M + H]^+^.

### 3.3. Synthesis of Amino-Modified Cyclic Pentapeptide Cyclo(-Arg[Pbf]-Gly-Asp[tBu]-D-Phe-Asp[PEG-amine]-)

Peptapeptide **1** (44 mg, 0.050 mmol, 1.0 eq), HOBt (10 mg, 0.075 mmol, 1.5 eq), HBTU (38 mg, 0.10 mmol, 2.0 eq), and DIPEA (41 µL, 0.25 mmol, 5.0 eq) were dissolved in 500 µL of DMF, and then 4,7,10-trioxa-1,13-tridecanediamine (220 µL, 1.0 mmol, 20 eq) was added. The mixture was stirred overnight at r.t. The reaction mixture was precipitated with diethyl ether, and the solid was dissolved in dichloromethane. The organic layer was washed with water and dried over Na_2_SO_4_. After concentration, the mixture was purified by column chromatography (silica gel:CH_2_Cl_2_-MeOH, 10:1) to produce amino-modified cyclic pentapeptide **2** as a light yellow solid in an 81% yield (44 mg, 0.041 mmol). ESI-HRMS: *m*/*z* = 1101.5633, calcd for C_52_H_81_N_10_O_14_S *m*/*z* = 1101.5654 [M + H]^+^.

### 3.4. Synthesis of Monomeric RGD Conjugate Pyro-MonoRGD

Pyro (4.9 mg, 0.009 mmol, 1.0 eq), HOBt (1.8 mg, 0.014 mmol, 1.5 eq) and EDC (3.5 mg, 0.018 mmol, 2.0 eq) were dissolved in 150 µL of DMF. DIPEA (7.4 µL, 0.045 mmol, 5.0 eq) and amino-modified pentapeptide **2** (10 mg, 0.009 mmol, 1.0 eq) were added. The mixture was reacted overnight in the dark. The reaction mixture was precipitated with diethyl ether. The solid was dissolved in dichloromethane and washed with water. The organic layer was dried (MgSO_4_), concentrated under reduced pressure, and purified via a silica gel column (CH_2_Cl_2_/MeOH = 15:1) to afford condensed compound **3** in an 80% yield (12.0 mg, 7.2 µmol). Deprotection was performed with 100 µL of the cleavage solution (TFA/Tis/water = 95:2.5:2.5) for 40 min at room temperature. The product was precipitated, washed three times with ethyl ether (0.5 mL), and purified with HPLC to produce Pyro-MonoRGD in a 71% yield (6.7 mg, 5.1 µmol). ESI-HRMS: *m*/*z* = 1309.6694, calcd for C_68_H_89_N_14_O_13_ *m/z* = 1309.6734 [M + H]^+^.

### 3.5. Synthesis of Tert-butyl (1,3-dihydroxypropan-2-yl)carbamate

2-Amino-1,3-propanediol (1.62 g, 17.8 mmol, 1.0 eq) was dissolved in 60 mL of tetrahydrofuran/H_2_O (1/1). Di-tert-butyl dicarbonate (Boc_2_O) (5.82 g, 26.7 mmol, 1.5 eq) and K_2_CO_3_ (6.15 g, 44.5 mmol, 2.5 eq) were added. The reaction was stirred at room temperature overnight. The reaction system was allowed to stand for stratification, and the aqueous phase was extracted three times with tetrahydrofuran. The organic phase was combined, dried over anhydrous Na_2_SO_4_, evaporated in vacuo, and purified by column chromatography (silica gel:petroleum ether/ethyl acetate, 1:1) to produce compound **4** as a white solid in a 62% yield (2.1 g, 11.0 mmol). ^1^H-NMR (400 MHz, CDCl_3_) δ 5.24 (s, 1H), 3.81 (qd, *J* = 11.1, 4.3 Hz, 4H), 3.69 (s, 1H), 2.50 (dd, *J* = 22.1, 14.7 Hz, 2H), 1.46 (s, 9H), which agrees with published data [39].

### 3.6. Synthesis of Di-tert-butyl-3,3′-((2-((tert-butoxycarbonyl)amino)propane-1,3-diyl)bis(oxy))dipropionate

Compound **4** (200 mg, 1.05 mmol, 1.0 eq) was dissolved in 500 µL of DMSO and treated with 5.0 M NaOH (21 µL, 0.105 mmol, 0.1 eq). Afterwards, tert-butyl acrylate (440 µL, 3.03 mmol, 2.9 eq) was added, and the solution was stirred at room temperature overnight. The reaction solution was diluted with ethyl ester, washed with H_2_O and then with saturated brine, and dried over anhydrous Na_2_SO_4_. After evaporation, the residue was purified by column chromatography (silica gel, petroleum ether/ethyl ester, 8:1), and compound **5** was obtained as a colorless oil in a 58% yield (273 mg, 0.61 mmol). ^1^H-NMR (400 MHz, CDCl_3_) δ 4.98 (d, *J* = 7.2 Hz, 1H), 3.81 (s, 1H), 3.71 – 3.60 (m, 4H), 3.52 (dd, *J* = 9.0, 3.5 Hz, 2H), 3.42 (dd, *J* = 9.3, 6.2 Hz, 2H), 2.45 (t, *J* = 6.3 Hz, 4H), 1.44 (s, 18H), 1.42 (s, 1H). ^13^C NMR (100.6 MHz, CDCl_3_): δ 170.89, 155.46, 80.53, 79.19, 77.38, 77.07, 76.75, 69.21, 66.81, 49.46, 36.27, 28.36, 28.08. LC-MS: *m*/*z* = 448.5, calcd for C_22_H_43_NO_8_ *m/z* = 448.3 [M + H]^+^.

### 3.7. Synthesis of Dimeric Linker 3,3′-((2-Aminopropane-1,3-diyl)bis(oxy))dipropionic acid hydrochloride

Compound **5** (134 mg, 0.30 mmol, 1.0 eq) was dissolved in 0.5 mL of dichloromethane, and hydrochloride in ethyl ether (2.8 M, 3 mL) was added. The reaction was allowed to proceed for 20 h at room temperature and then concentrated under reduced pressure. Dimeric linker **6** was obtained as a white solid in a 93% yield (75 mg, 0.28 mmol). ^1^H-NMR (300 MHz, D_2_O) δ 3.93–3.76 (m, 6H), 3.73 (dd, *J* = 9.4, 6.1 Hz, 3H), 2.73 (t, *J* = 5.8 Hz, 4H). ^13^C NMR (100.6 MHz, D_2_O): δ 176.33, 67.21, 66.50, 50.54, 34.23. LC-MS: *m*/*z* = 236.4, calcd for C_9_H_18_NO_6_ *m*/*z* = 236.1 [M + H]^+^.

### 3.8. Synthesis of Pyro-Conjugated Dimeric Linker 7

A mixture of Pyro (32 mg, 0.06 mmol, 1.0 eq), NHS (70 mg, 0.6 mmol, 10 Equation), and EDC (115 mg, 0.6 mmol, 10 eq) was stirred in DMF (5 mL) at r.t. for 20 h without light. The reaction mixture was diluted with ethyl acetate, washed with water and saturated salt water, and dried with Na_2_SO_4_. After evaporation, the activated ester was obtained. The activated ester (10 mg, 0.016 mmol, 1.0 eq) was dissolved in 300 µL of DMF, and compound **6** (5 mg, 0.019 mmol, 1.2 eq) and DIPEA (13 µL, 0.08 mmol, 5.0 eq) were added. The reaction solution was diluted with water, the pH was adjusted with 1.0 M HCl, and then it was extracted with EtOAc. The organic layer was combined and evaporated, and the residue was purified via silica gel (DCM/MeOH = 10:1) to produce Pyro-conjugated dimeric linker **7** as a black solid in a 92% yield (11.0 mg, 0.055 mmol). ^1^H-NMR (300 MHz, CDCl_3_) δ 9.23 (s, 1H), 9.15 (s, 1H), 8.43 (s, 1H), 8.00 (s, 3H), 7.86 (dd, *J* = 17.8, 11.5 Hz, 1H), 6.64 (d, *J* = 8.1 Hz, 1H), 6.26–6.07 (m, 2H), 5.47 (d, *J* = 20.3 Hz, 1H), 5.05 (d, *J* = 20.2 Hz, 1H), 3.47 (s, 3H), 3.33 (s, 3H), 3.14 (s, 3H), 2.96 (s, 8H), 2.89 (d, *J* = 0.6 Hz, 9H), 2.65 (s, 1H), 2.07 (s, 1H), 1.84 (d, *J* = 7.2 Hz, 3H), 1.63 (t, *J* = 7.6 Hz, 3H), 1.27 (s, 3H). HRMS-ESI: *m*/*z* = 752.3651, calcd for C_42_H_50_N_5_O_8_ *m*/*z* = 752.3659 [M + H]^+^.

### 3.9. Synthesis of Conjugate Pyro-DiRGD

Pyro-conjugated dimeric linker **7** (2.3 mg, 0.003 mmol, 1.0 eq), HOBt (1.8 mg, 0.013 mmol, 4.4 eq), and HBTU (5.0 mg, 0.013 mmol, 4.4 eq) were dissolved in 50 µL of DMF. Then, DIPEA (5.5 µL, 0.033 mmol, 11 eq) and amino-modified RGD peptide **2** (8.0 mg, 0.0073 mmol, 2.4 eq) were added. The mixture was reacted overnight in the dark. The reaction mixture was precipitated with diethyl ether. The solid was dissolved in dichloromethane and washed with brine. The organic layer was dried (MgSO_4_), concentrated under reduced pressure, and purified via silica gel (DCM/MeOH = 10:1) to afford protected conjugate **8** in a 72% yield (6.5 mg, 0.002 mmol). Deprotection was performed with 100 µL of the cleavage solution (TFA/Tis/water = 95:2.5:2.5) for 40 min at room temperature. The product was precipitated, washed three times with anhydrous ether (0.5 mL) and purified with HPLC to produce Pyro-DiRGD in a 67% yield (3.2 mg, 1.4 µmol). HRMS-ESI: *m*/*z* = 1151.0880, calcd for C_112_H_159_N_25_O_28_ *m*/*z* = 1151.0893 [M + 2H]^2+^/2.

### 3.10. Synthesis of Tris((2-(tert-butoxycarbonyl)ethoxyl)methyl)methylamine

Tris(hydroxymethyl)aminomethane (2.4 g, 0.02 mol, 1.0 eq) was dissolved in DMSO (4 mL) and treated with NaOH (0.4 mL, 5 M). Afterwards, tert-butylacrylate (9.0 g, 0.07 mol, 3.5 eq) was added. The opaque solution was stirred at room temperature for 20 h. Water was added to the reaction mixture, and it was extracted with dichloromethane three times and then washed with brine. The organic layer was dried (Na_2_SO_4_), concentrated, and purified by silica gel column chromatography (petroleum ether/ethyl ester, 1:2) to afford compound **9** as a pale yellow oil in a 38% yield (3.86 g, 7.63 mmol). ^1^H-NMR (400 MHz, CDCl_3_): δ 3.60 (t, *J* = 6.4 Hz, 6H), 3.27 (s, 6H), 2.41 (t, *J* = 6.4 Hz, 6H), 1.40 (s, 27H), which agrees with the published data [40].

### 3.11. Synthesis of Pyro-Conjugated Compound

Compound **9** (100 mg, 0.197 mmol, 1.0 eq) was dissolved in DMF (5 mL). Afterwards, EDC (77 mg, 0.4 mmol, 2.0 eq) and DIPEA (165 µL, 1 mmol, 5.0 eq) were added to the solution followed by the addition of Pyro (105 mg, 0.197 mmol, 1.0 eq). The solution was stirred overnight at RT. The reaction mixture was diluted with dichloromethane, washed with water and brine and dried with Na_2_SO_4_. After concentration, the residue was purified by silica gel column chromatography (CH_2_Cl_2_/MeOH = 50:1) to afford compound **10** as a blue-purple solid in a 90% yield (180 mg, 0.176 mmol). ^1^H-NMR (400 MHz, CDCl_3_) δ 9.51 (s, 1H), 9.40 (s, 1H), 8.56 (s, 1H), 8.02 (dd, *J* = 17.8, 11.5 Hz, 1H), 6.23 (dd, *J* = 47.3, 14.6 Hz, 3H), 6.04 (s, 1H), 5.21 (dd, *J* = 88.1, 19.9 Hz, 3H), 4.55 (d, *J* = 6.7 Hz, 1H), 4.33 (d, *J* = 8.7 Hz, 1H), 3.58 (t, *J* = 6.3 Hz, 6H), 3.41 (s, 3H), 3.25 (s, 3H), 2.36 (t, *J* = 6.2 Hz, 6H), 1.82 (d, *J* = 7.2 Hz, 3H), 1.70 (t, *J* = 7.6 Hz, 3H), 1.57 (s, 8H), 1.27 (s, 27H), 0.45 (s, 1H), −1.69 (s, 2H). ^13^C NMR (CDCl_3_, 100.6 MHz): δ 196.35, 172.41, 171.72, 170.91, 161.02, 154.96, 150.58, 148.96, 144.81, 141.34, 137.75, 136.01, 135.90, 135.63, 131.40, 130.50, 129.21, 128.16, 122.39, 106.12, 103.85, 96.95, 93.11, 80.30, 77.32, 77.20, 77.00, 76.68, 69.08, 66.95, 59.68, 51.70, 49.83, 48.06, 36.01, 33.63, 30.35, 28.07, 27.89, 23.19, 19.41, 17.43, 12.09, 12.00, 11.19. ESI-HRMS: *m*/*z* = 1022.5861, calcd for C_58_H_80_N_5_O_11_ *m*/*z* = 1022.5854 [M + H]^+^.

### 3.12. Synthesis of Deprotected Pyro-Conjugated Trimeric Linker

Trifluoroacetic acid (TFA) (2.0 mL) was added to the dichloromethane (2.0 mL) solution of compound **10** (100 mg, 0.098 mmol, 1.0 eq). The mixture was stirred overnight at room temperature and concentrated under reduced pressure. Then, the residues were dissolved in dichloromethane, washed with water, and dried with MgSO_4_. Concentration under reduced pressure afforded deprotected Pyro-conjugated trimeric linker **11** as a purple solid in an 85% yield (75 mg, 0.088 mmol). ^1^H-NMR (400 MHz, CDCl_3_) δ 9.95 (s, 1H), 9.76 (s, 1H), 8.87 (s, 1H), 7.87 (dd, *J* = 17.6, 11.8 Hz, 1H), 6.59 (s, 1H), 6.25 (dd, *J* = 14.5, 9.7 Hz, 2H), 5.41 (d, *J* = 19.9 Hz, 1H), 5.16 (d, *J* = 19.7 Hz, 1H), 3.74–3.59 (m, 8H), 3.58–3.44 (m, 10H), 3.32 (s, 6H), 2.32 (d, *J* = 4.9 Hz, 7H), 1.85 (d, *J* = 4.9 Hz, 3H), 1.74 (t, *J* = 7.3 Hz, 3H), 1.25 (s, 5H), 0.84 (d, *J* = 4.0 Hz, 4H). ESI-HRMS: *m*/*z* = 854.3974, calcd for C_46_H_55_N_5_O_11_ *m*/*z* = 854.3976 [M + H]^+^.

### 3.13. Synthesis of the Trimeric Conjugate Pyro-TriRGD

Pyro-conjugated trimeric linker **11** (1.2 mg, 0.0014 mmol, 1.0 eq), HOBt (1.2 mg, 0.009 mmol, 6.6 eq), and HBTU (3.5 mg, 0.009 mmol, 6.6 eq) were dissolved in 50 µL of DMF. DIPEA (3.8 µL, 0.023 mmol, 16.5 eq) and amino-modified RGD peptide **2** (5.0 mg, 0.0046 mmol, 3.3 eq) were added. The mixture was reacted overnight in the dark. The reaction mixture was precipitated with diethyl ether, and the solid was dissolved in dichloromethane and washed with water. The organic layer was dried (MgSO_4_) and concentrated under reduced pressure. The residue was purified using a silica gel column (CH_2_Cl_2_/MeOH = 10:1) to afford protected conjugate **12** in a 62% yield (3.6 mg, 0.0009 mmol). Deprotection was performed with 100 µL of the cleavage solution (TFA/Tis/water = 95:2.5:2.5) for 40 min at room temperature. The product was precipitated, washed three times with ethyl ether (0.5 mL), and purified with HPLC to produce Pyro-TriRGD in a 70% yield (3.0 mg, 0.97 µmol). ESI-HRMS: *m*/*z* = 1059.8714, calcd for C_151_H_219_N_35_O_41_ *m*/*z* = 1059.8735 [M + 3H]^3+^/3.

### 3.14. Determination of the Photophysical and Photochemical Properties

The UV–Vis absorption spectra and fluorescence spectra of the synthesized conjugates of Pyro-MonoRGD, Pyro-DiRGD, Pyro-TriRGD, and free Pyro were measured in DMSO. Absorption spectra were recorded from 300 to 800 nm. Fluorescence emission and excitation spectra were recorded from 550 to 800 nm upon excitation at approximately 668 nm and emission at 672–673 nm using a Hitachi model F-4500 FL spectrophotometer (Tokyo, Japan). The singlet oxygen quantum yields (Φ_Δ_) of the conjugates were measured in DMSO using DPBF as the quencher and Pyro as the reference.

### 3.15. Receptor Binding Assay

Human integrin α_v_β_3_ (ITGAV&ITGB3) heterodimer protein was diluted to 2.0 μg/mL in PBS buffer (pH 7.4) and was then added to a 96-well plate (100 μL/well) and incubated overnight at 4 °C. The plate was blocked with 10% milk solution for an additional 2 h in a 37 °C shaker, followed by a 3 h incubation in the 37 °C shaker with various concentrations (10^−3^ to 10^−11^ M) of test compounds in the presence of biotinylated vitronectin (2.0 μg/mL). After washing, the plates were incubated for 1 h in the 37 °C shaker with streptavidin–HRP and then washed again, followed by 30 min of incubation with 100 μL/well TMB single-component substrate solution before stopping the reaction with the addition of 100 μL/well 2.0 N H_2_SO_4_. The absorbance at 450 nm was read on a microplate reader. Each data point represents the average of the triplicate wells, and data analysis was performed using nonlinear regression analysis with GraphPad Prism 5.0 software (GraphPad Software, Inc., San Diego, CA, USA.).

### 3.16. In Vivo Tumor Enrichment Analysis

Human U87-MG cells (4 × 10^6^ cells/mouse) were subcutaneously inoculated on the axilla of female Balb/c nude mice. When the tumors grew to 200 mm^3^, the mice were divided into three groups with each group containing three mice. The mice were then intravenously injected with 50 nmol/mouse of Pyro-MonoRGD, Pyro-DiRGD, and Pyro-TriRGD, respectively. In vivo fluorescence analysis was performed before and after the injection at various time points using the IVIS Lumina imaging system (IVIS Lumina II, Xenogen, USA) with a Cy5.5 filter (excitation: 615–665 nm, emission: 695–770 nm). Alternatively, the mice were euthanized at 2 h postinjection, and major organs, including the heart, liver, spleen, lungs, kidneys, and muscles, as well as the tumor, were collected; fluorescence images were then acquired.

## 4. Conclusions

To improve the tumor enrichment of small molecule ligand-conjugated photosensitizers, we designed and synthesized multivalent cyclic RGD peptide-conjugated Pyro molecules. These conjugates were prepared using a convergent strategy, and the synthesis was carefully characterized. Their UV–Vis absorption spectra, fluorescence spectra, and singlet oxygen quantum yields were measured. The subsequent binding affinity assay verified the improvement in the binding towards the integrin α_v_β_3_ receptors after the increase in the valence, which was a nearly 20-fold improvement in the binding affinity of Pyro-TriRGD compared with that of monomeric conjugate Pyro-MonoRGD. The in vivo distribution experiments on the tumor-bearing mice also confirmed a significant increase in the distribution of the conjugates in the tumor sites via multimerization, where the trimeric conjugate, Pyro-TriRGD, had the best tumor enrichment compared with the other two conjugates. These results indicated that the multivalence interaction may be used to increase the tumor enrichment of RGD peptide-conjugated Pyro photosensitizers for tumor targeting PDT, and the trimeric conjugate Pyro-TriRGD may be used as a novel antitumor photodynamic agent with high tumor enrichment.

## Figures and Tables

**Table 1:** Photophysical and photochemical data for Pyro-MonoRGD, Pyro-DiRGD, Pyro-TriRGD, and Pyro in DMSO.

Compound	λ_max_ (nm)	λ_em_ (nm)	Stokes Shift (nm)	Φ_Δ_
Pyro	668	673	5	0.52
Pyro-MonoRGD	668	672	4	0.49
Pyro-DiRGD	668	673	5	0.47
Pyro-TriRGD	668	673	5	0.48

**Table 2:** Inhibition of biotinylated vitronectin binding to α_v_β_3_ receptors. IC_50_ values were calculated as the concentration of compound required for 50% inhibition of biotinylated vitronectin binding as estimated by GraphPad Prism 5.0. All values are the means (± standard deviation) of triplicate determinations.

Compounds	Pyro-TriRGD	Pyro-DiRGD	Pyro-MonoRGD
IC_50_ (nM)	43.8 ± 8.2	313.2 ± 23.1	950.7 ± 39.6

## Supplementary Materials

The following are available online. HPLC analysis, Mass spectrometry analysis, and UV-Vis and fluorescence spectrum analysis of the reported conjugates, NMR spectra of the intermediates.
