# Supplementary material for: Multimerization Increases Tumor Enrichment of Peptide–Photosensitizer Conjugates
Source: Molecules. 2019 Feb 25;24(4):817. doi: 10.3390/molecules24040817 (PMC6413024; doi:10.3390/molecules24040817)
Supplement: Supplementary file 1 [file molecules-24-00817-s001.pdf]

# Supporting information

## 1. HPLC analysis of compounds.

HPLC analysis was performed using a C-18 column (150 × 4.6 mm) with a 0.6 mL/min flow rate and checked with a detector (λ = 380 or 220 nm). The column was initially held at 10% $CH_3CN$ - 90% $H_2O$. The concentration of $CH_3CN$ was ramped to 100% in 25 min and this concentration was maintained for 5 min and returned to 10% within 9 min. Before each next injection, the column was allowed to equilibrate to the initial mobile phase conditions for 30 min.

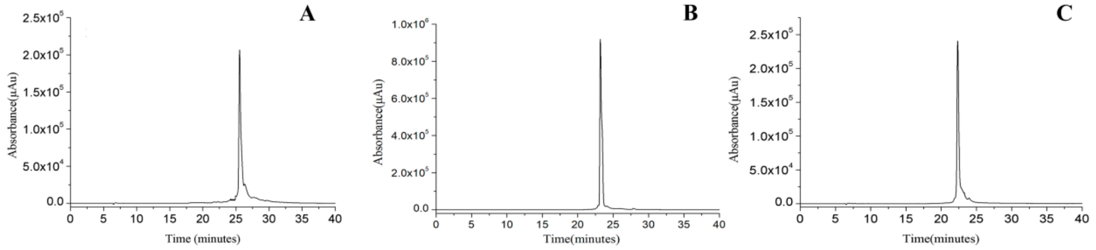


Fig. S1. HPLC traces (λ = 380 nm) of the conjugates (A) Pyro-MonoRGD, (B) Pyro-DiRGD and (C) Pyro-TriRGD.

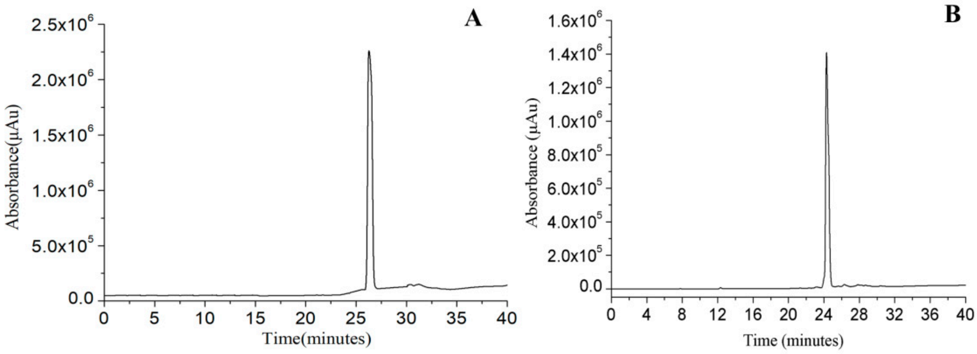


Fig. S2. HPLC traces (λ = 220 nm) of (A) cyclic RGD pentapeptide **1** and (B) amino-modified cyclic RGD peptapeptide **2** after purification.

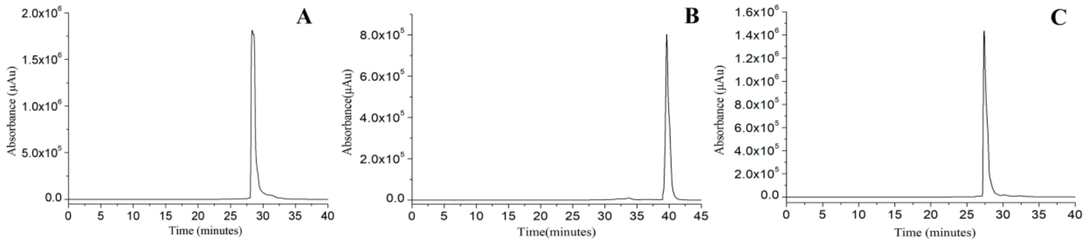


Fig. S3. HPLC traces (λ = 380 nm) of (A) Pyro-conjugated dimeric linker **7**, (B) compound **10** and (C) Pyro-conjugated trimeric linker **11** after purification.

**2. Mass spectrometry analysis of compounds.**

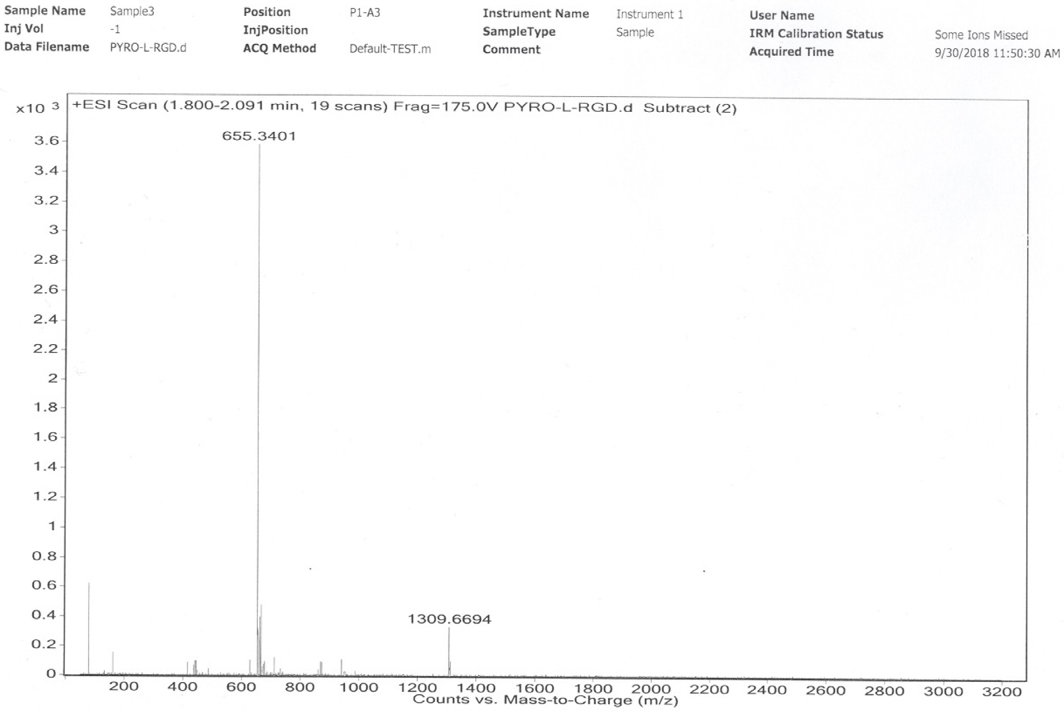


Fig. S4. Mass spectrometry analysis of Pyro-MonoRGD (ESI-HRMS: *m/z* = 1309.6694, calcd for $C_{68}H_{89}N_{14}O_{13}$ *m/z* = 1309.6734 $[M+H]^+$).

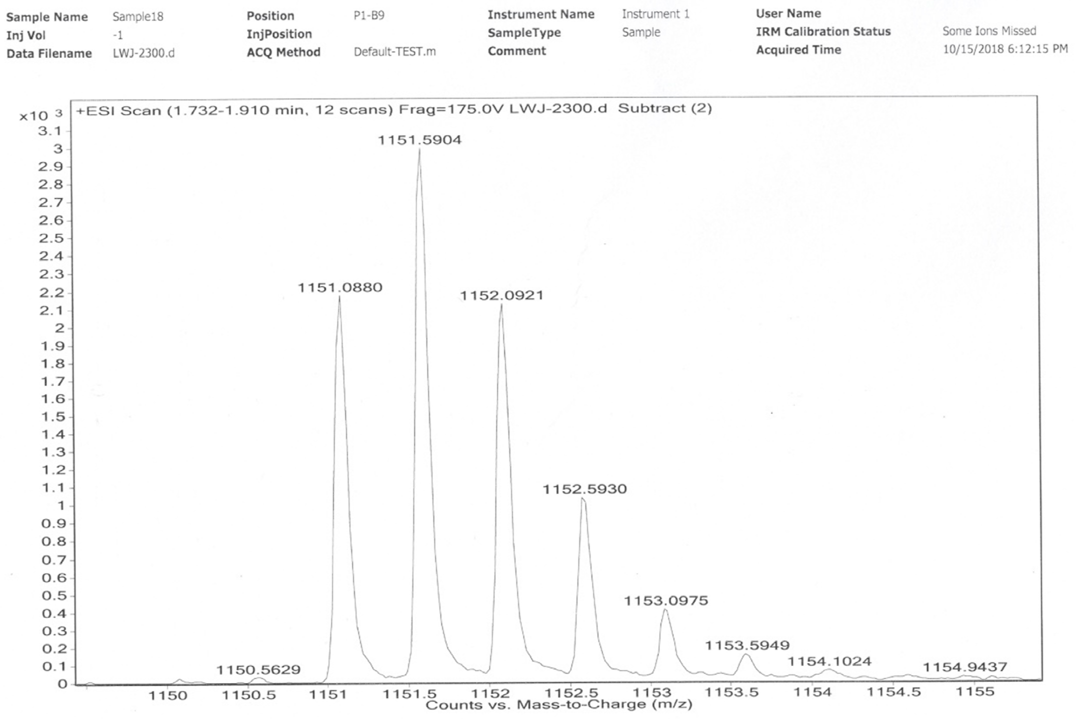


Fig. S5. Mass spectrometry analysis of Pyro-DiRGD (HRMS-ESI: *m/z* = 1151.0880, calcd for $C_{112}H_{159}N_{25}O_{28}$ *m/z* = 1151.0893 $[M+2H]^{2+}/2$).

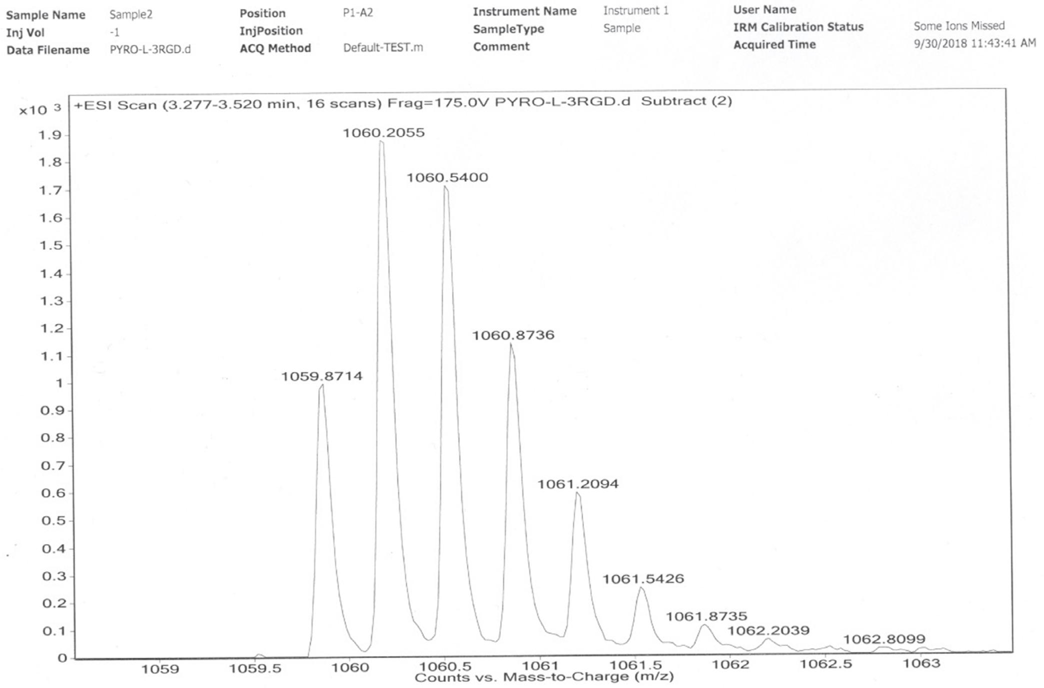


Fig. S6. Mass spectrometry analysis of Pyro-TriRGD (ESI-HRMS: *m/z* = 1059.8714, calcd for $C_{151}H_{220}N_{35}O_{41}$ *m/z* = 1059.8735 $[M + 3H]^{3+}/3$).

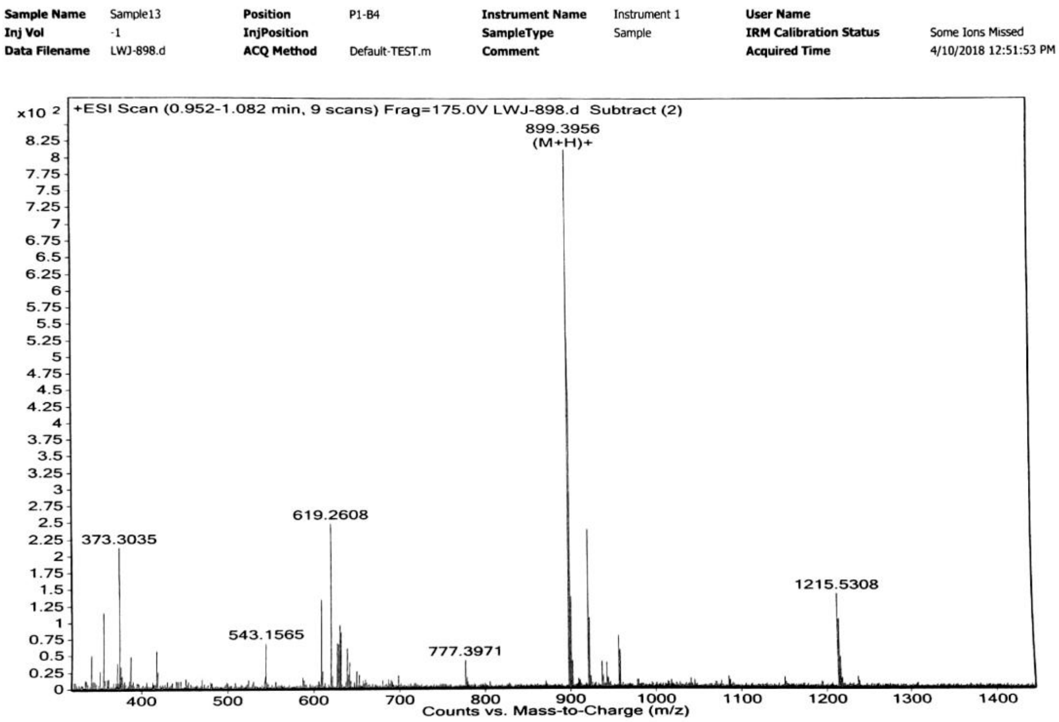


Fig. S7. Mass spectrometry analysis of protected pentapeptide cyclo(-Arg[Pbf]GlyAsp[tBu]-D-Phe-Asp-) **1** (HRMS-ESI: *m/z* = 899.3956, calcd for $C_{42}H_{59}N_8O_{12}S$ *m/z* = 899.3973 $[M+H]^+$).

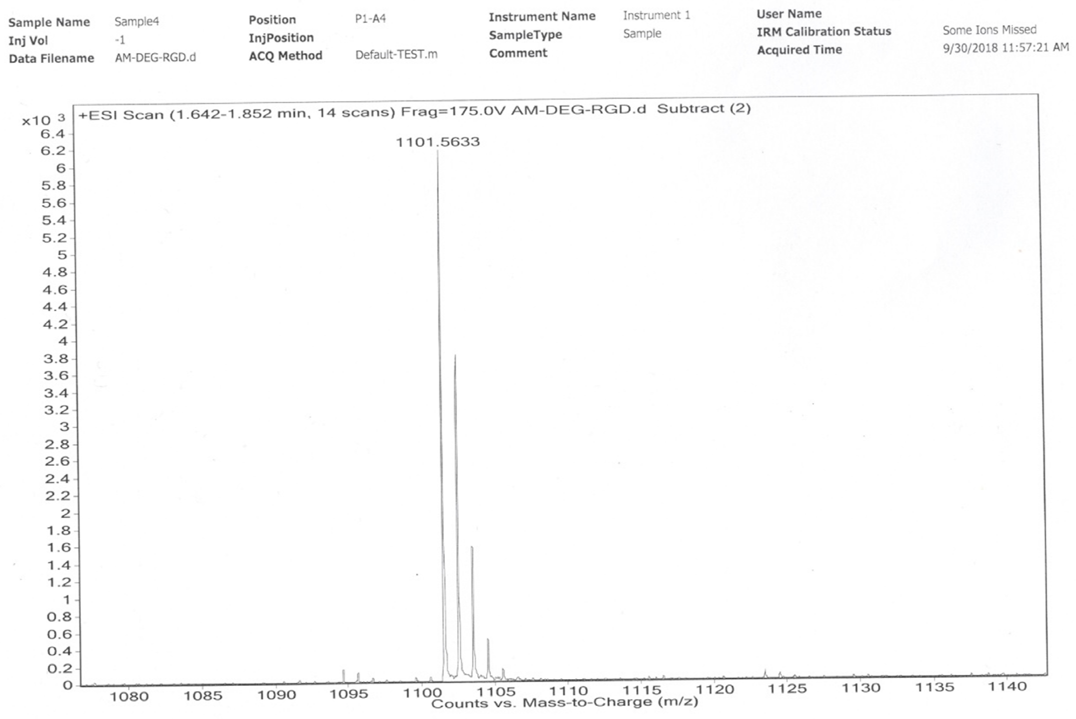


Fig. S8. Mass spectrometry analysis of amino-modifed cyclic pentapeptide **2** (ESI-HRMS: *m/z* = 1101.5633, calcd for $C_{52}H_{81}N_{10}O_{14}S$ *m/z* = 1101.5654 $[M+H]^+$).

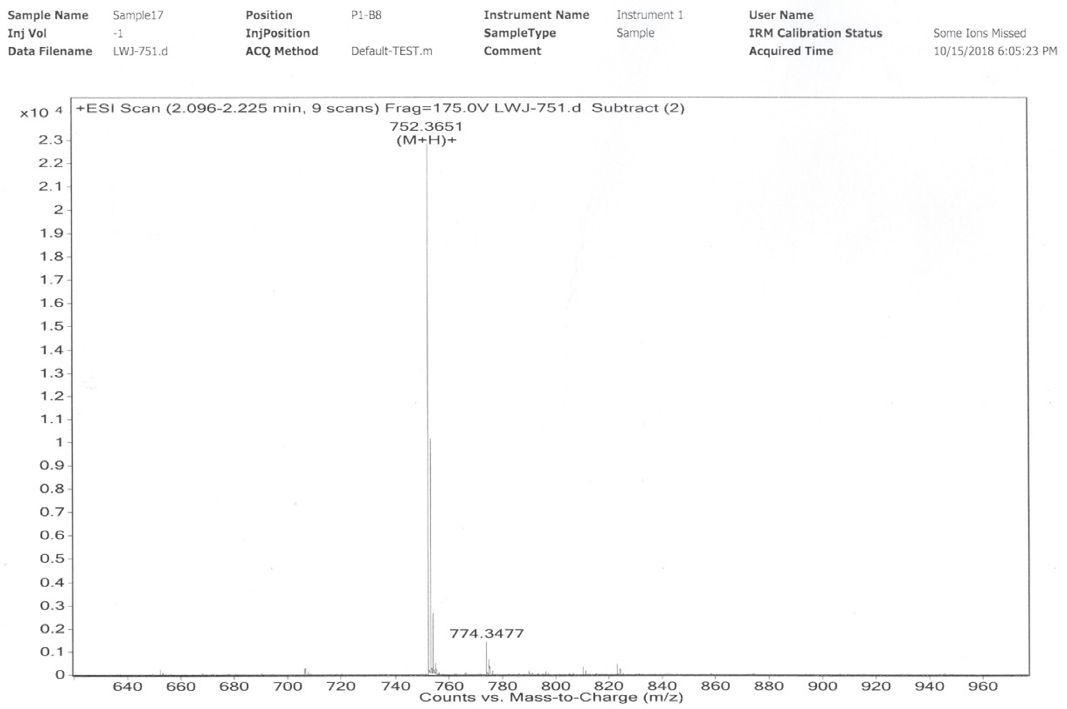


Fig. S9. Mass spectrometry analysis of Pyro-conjugated dimeric linker **7** (HRMS-ESI: *m/z* = 752.3651, calcd for $C_{42}H_{50}N_5O_8$ *m/z* = 752.3659 $[M+H]^+$).

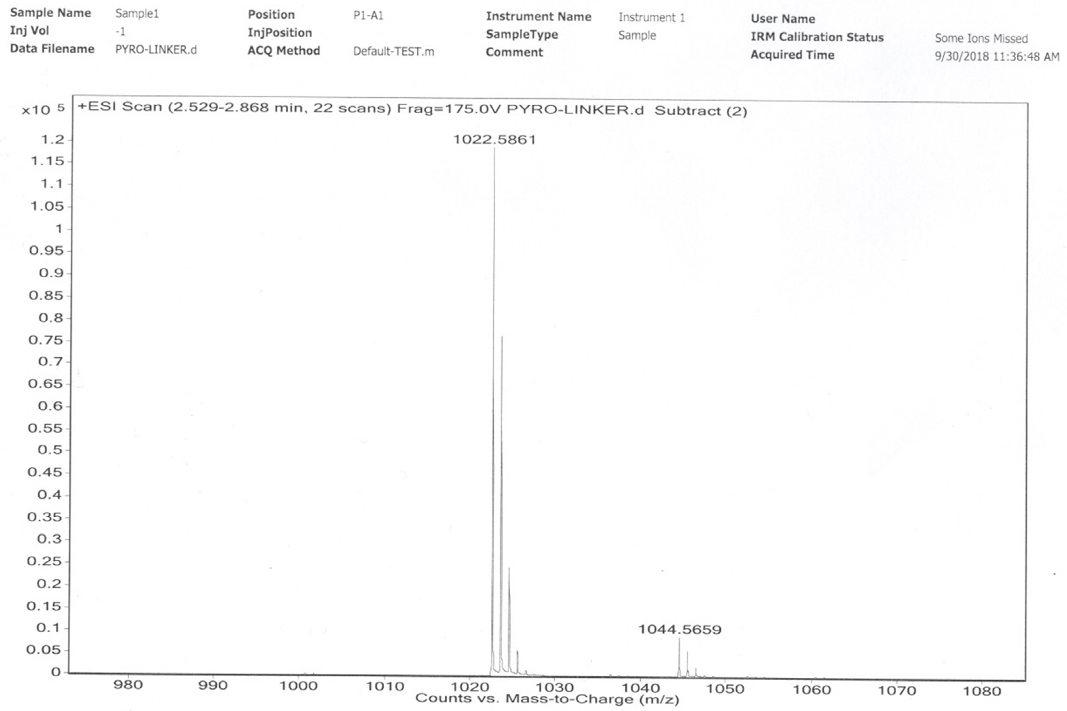


Fig. S10. Mass spectrometry analysis of compound **10** (ESI-HRMS: *m/z* = 1022.5861, calcd for $C_{58}H_{80}N_5O_{11}$ *m/z* = 1022.5854 $[M+H]^+$).

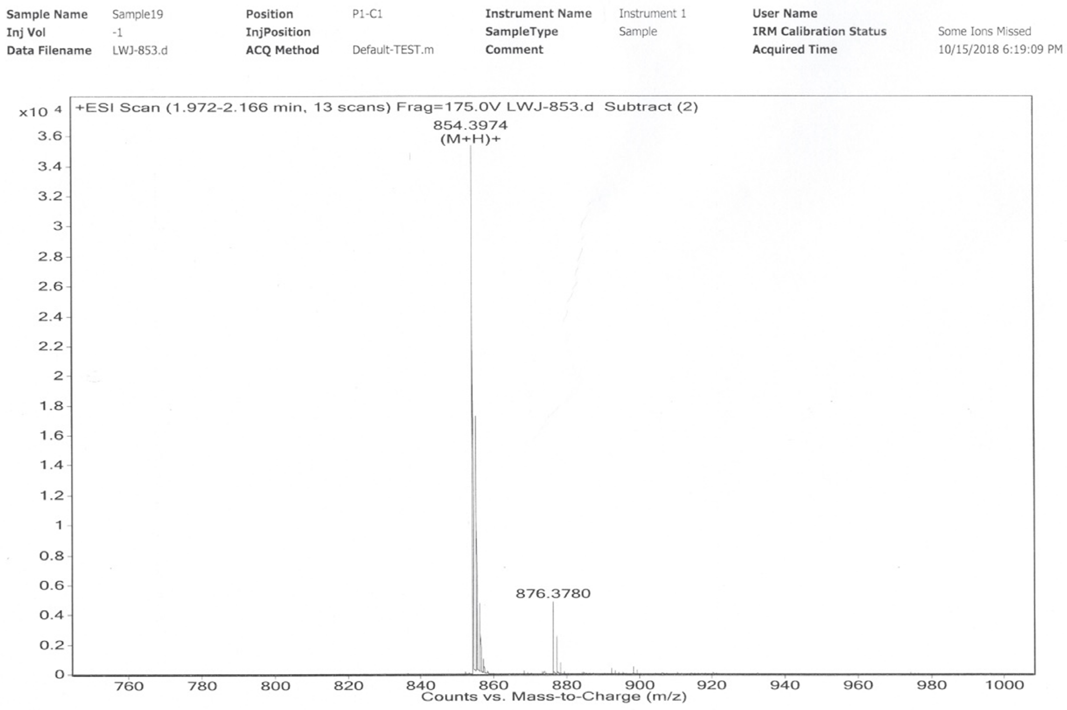


Fig. S11. Mass spectrometry analysis of Pyro-conjugated trimeric linker **11** (ESI-HRMS: *m/z* = 854.3974, calcd for $C_{46}H_{56}N_5O_{11}$ *m/z* = 854.3976 $[M+H]^+$).

**3. $^{1}H$ and $^{13}C$ NMR spectra of compounds.**

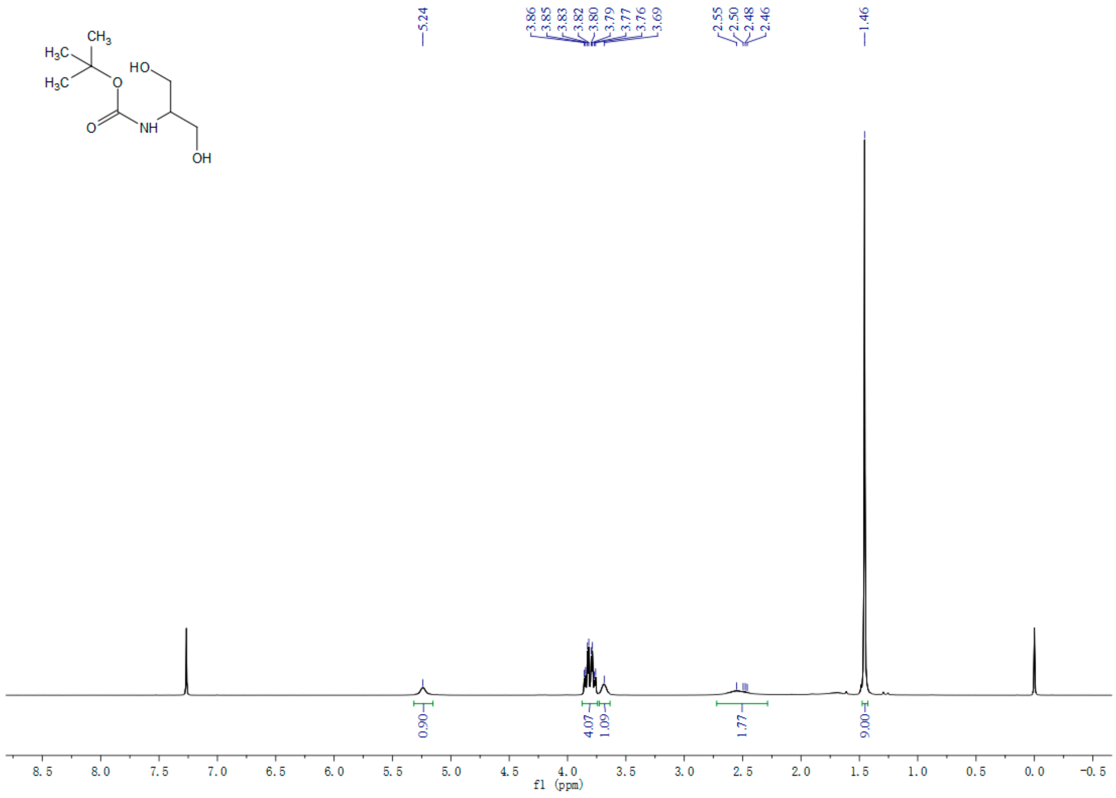


Fig. S12. $^{1}H$ NMR spectrum (400 MHz, $CDCl_3$) of compound **4**.

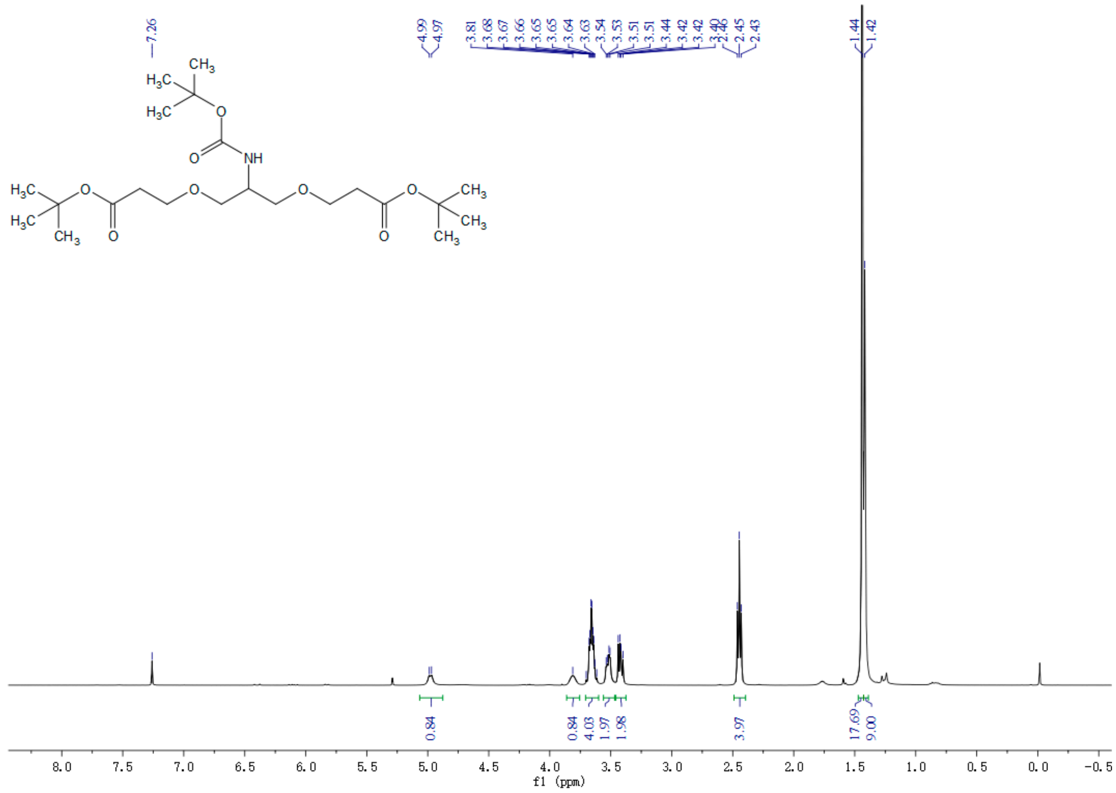


Fig. S13. $^{1}$H NMR spectrum (400 MHz, $CDCl_3$) of compound **5**.

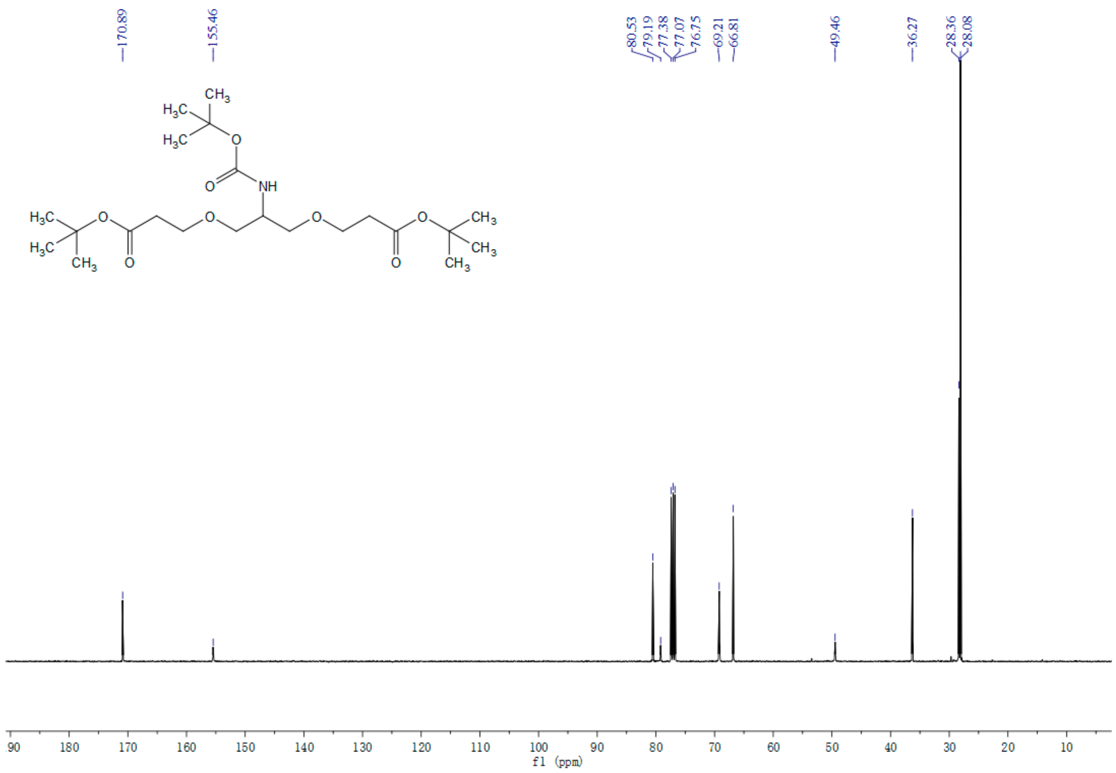


Fig. S14. $^{13}$C NMR spectrum (100.6 MHz, $CDCl_3$) of compound **5**.

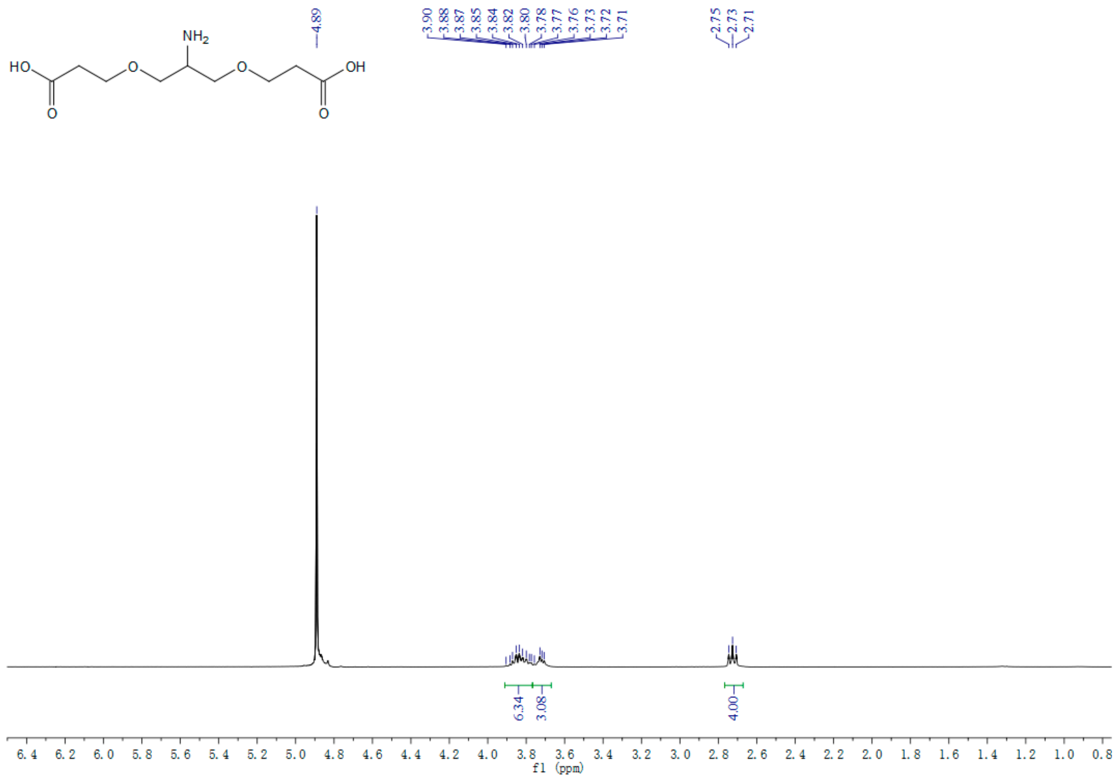


Fig. S15. $^{1}$H NMR spectrum (300 MHz, $D_2O$) of dimeric linker **6**.

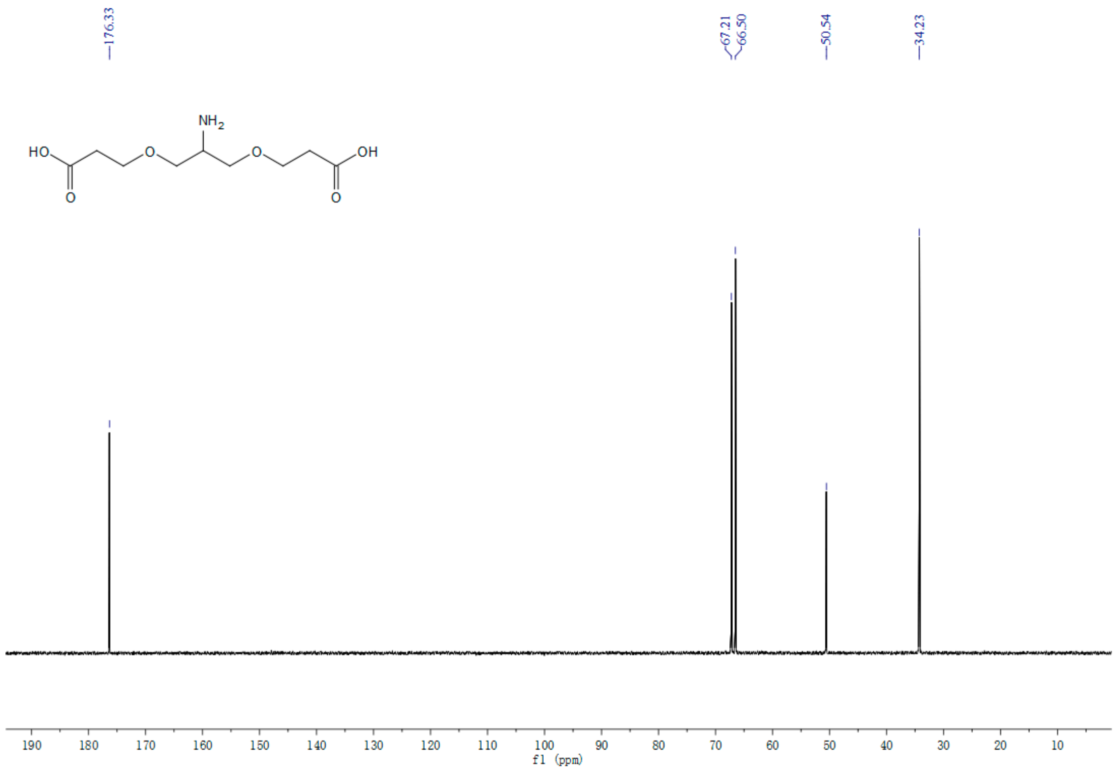


Fig. S16. $^{13}C$ NMR spectrum (100.6 MHz, $D_2O$) of dimeric linker **6**.

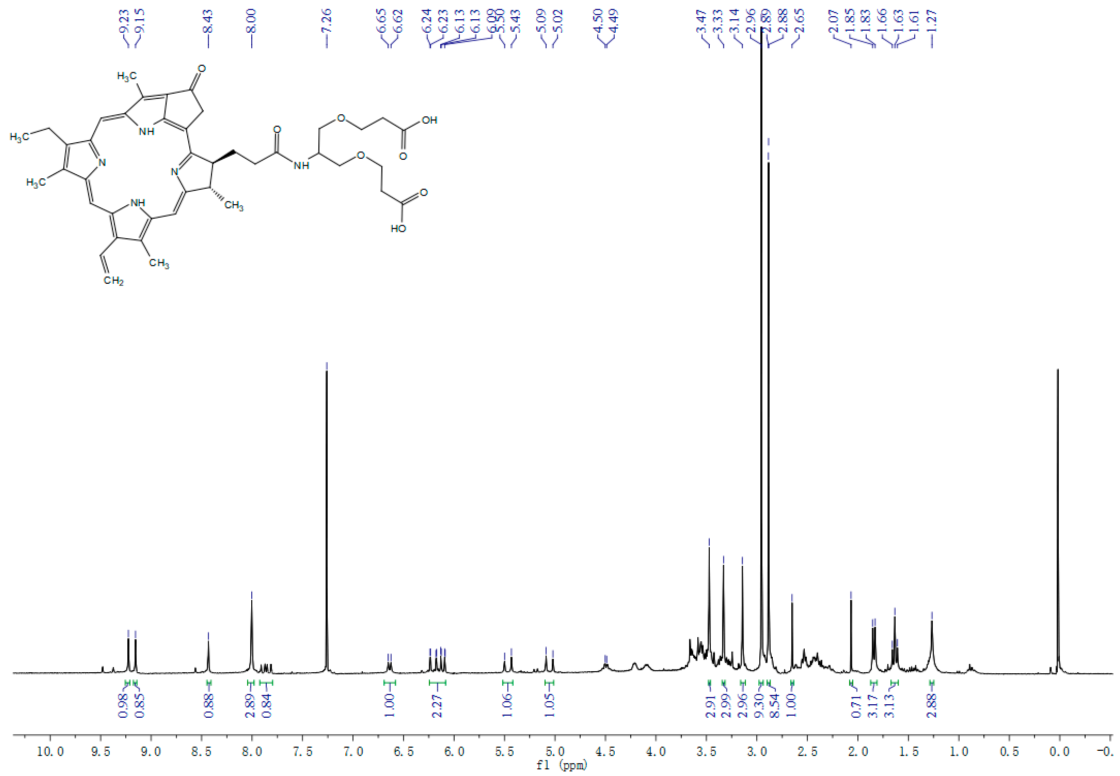


Fig. S17. $^{1}$H NMR spectrum (300 MHz, $CDCl_3$) of Pyro-conjugated dimeric linker **7**.

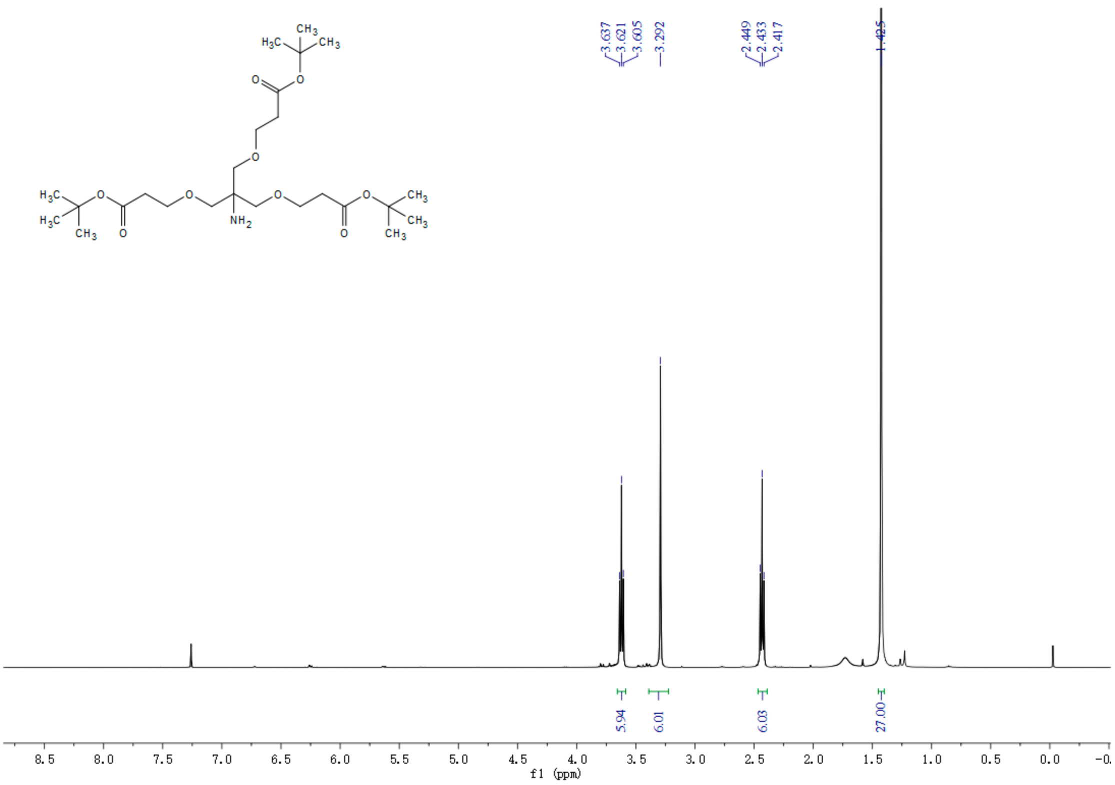


Fig. S18. $^{1}$H NMR spectrum (400 MHz, $CDCl_3$) of compound **9**.

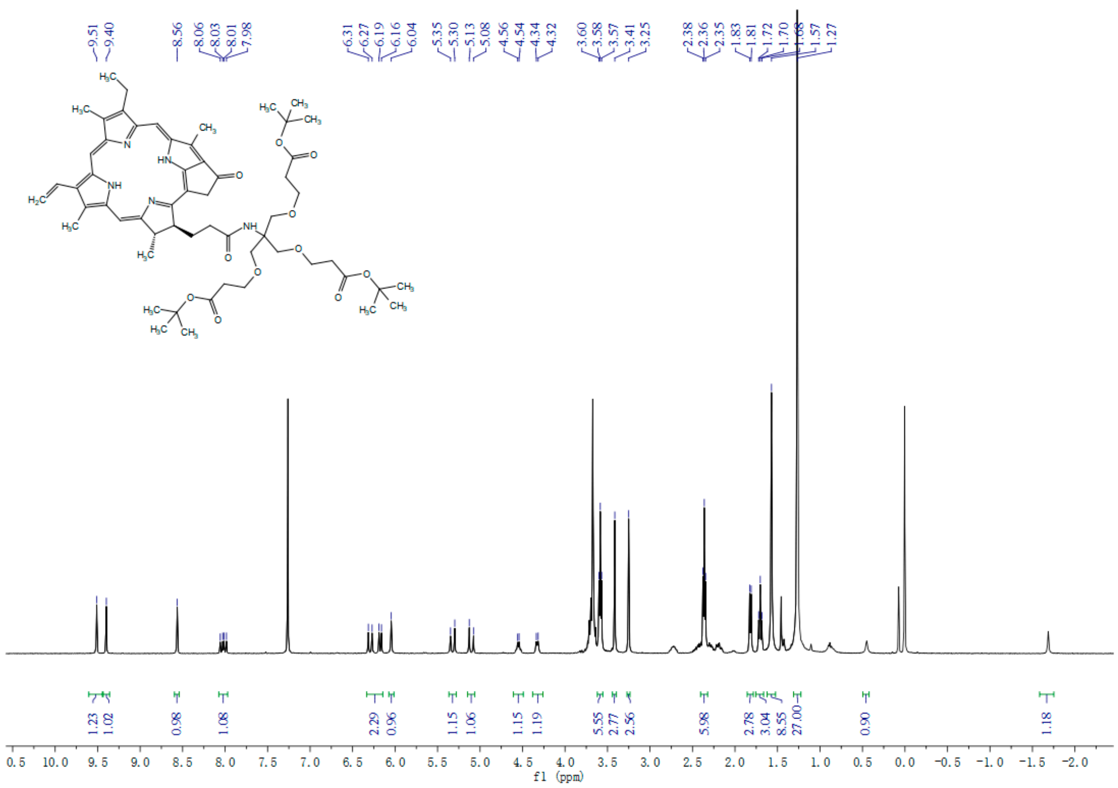

Fig. S19. $^1H$ NMR spectrum (400 MHz, $CDCl_3$) of compound **10**.

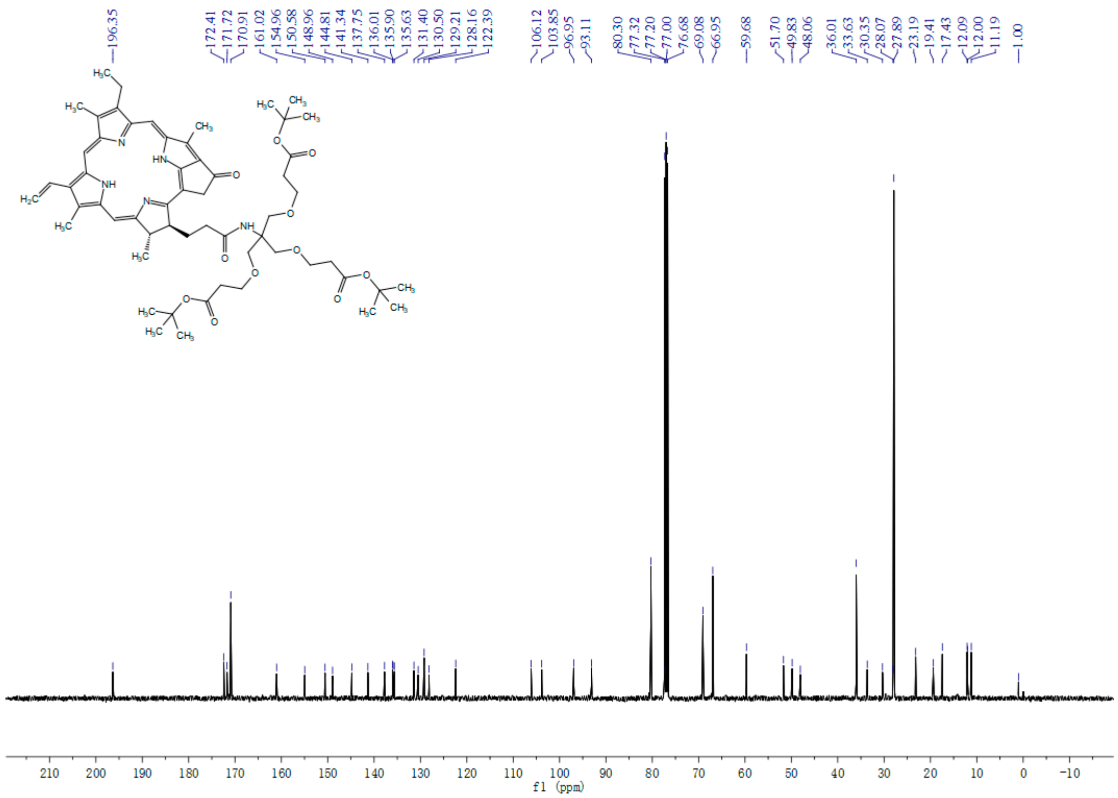

Fig. S20. $^{13}C$ NMR spectrum (100.6 MHz, $CDCl_3$) of compound **10**.

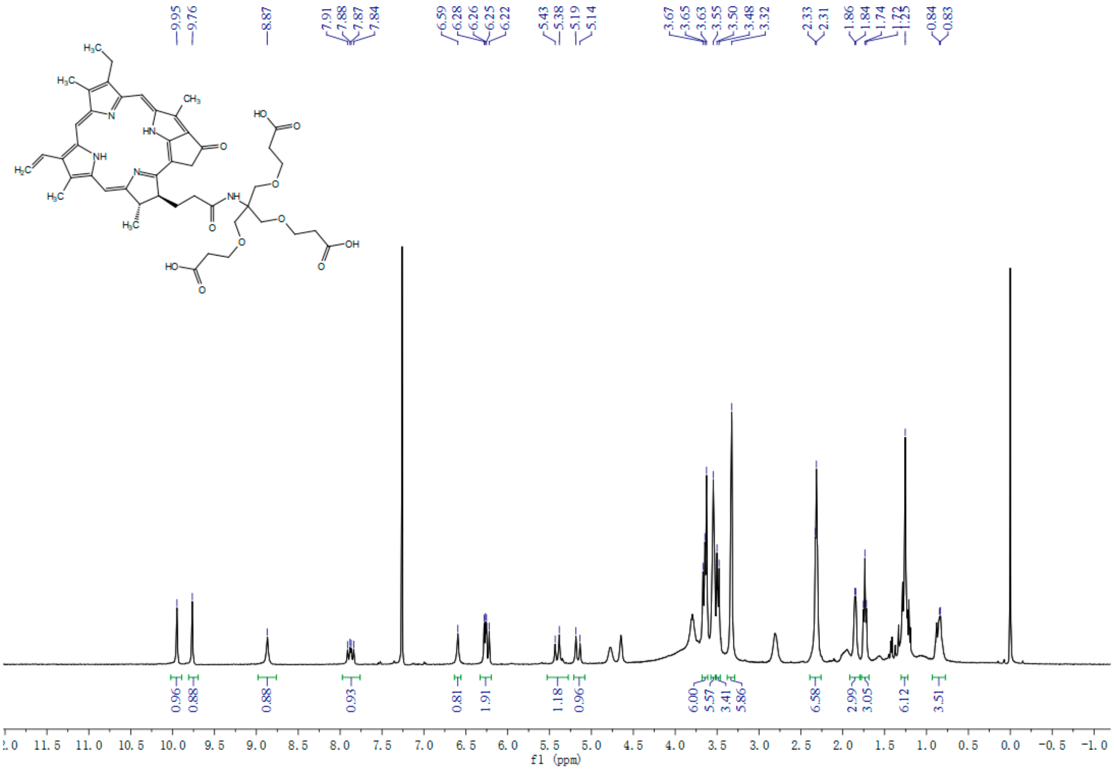

Fig. S21. ¹H NMR spectrum (400 MHz, $CDCl_3$) of Pyro-conjugated trimeric linker **11**.

## 4. The detection of singlet oxygen quantum yield in DMSO.

The UV absorption spectra of the photosensitizers in the wavelength range from 300 nm to 800 nm were measured with DPBF ($3 \times 10^{-5}$ mol · $L^{-1}$) and irradiated with the light of 680 nm. The absorbance at 680 nm was recorded, and the photobleaching rate of DPBF at 417 nm was recorded for different durations of light irradiation. The singlet oxygen quantum yield was obtained according to the following equation:

$$\Phi_{\Delta}=\Phi_{\Delta}^{Std}\frac{R_{DPBF}^{s.m.}(1-10^{-A_{680}^{Std}})}{R_{DPBF}^{Std}(1-10^{-A_{680}^{s.m.}})}$$

Where $\Phi_{\Delta}^{Std}$ is the singlet oxygen quantum yield of Pyro in DMSO, $\Phi_{\Delta}^{Std}$ = 0.52. $R_{DPBF}^{s.m.}$ and $R_{DPBF}^{Std}$ are photobleaching rates at 417 nm for each photosensitizer, respectively. $A_{680}^{Std}$ and $A_{680}^{s.m.}$ represent the absorbance of Pyro and compounds at 680 nm, respectively.

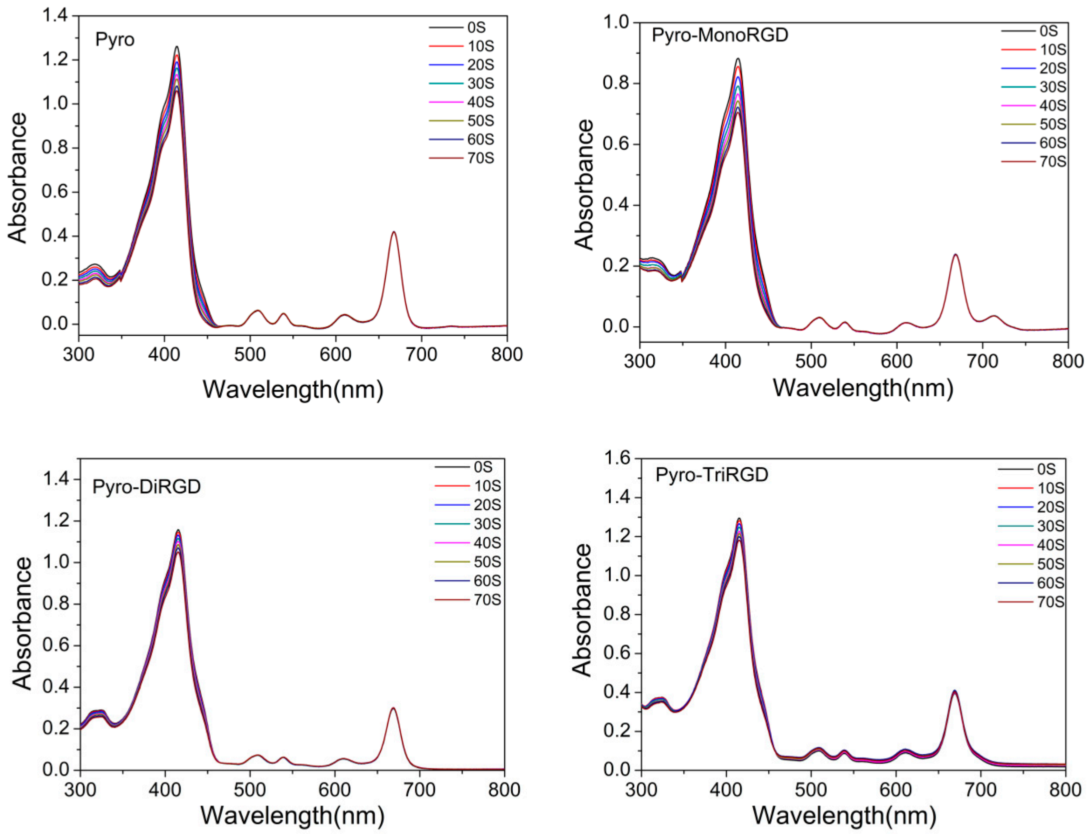


Fig. S22. singlet oxygen quantum yield data in DMSO. Typical spectra for determination of the singlet oxygen quantum yield of free Pyro, Pyro-MonoRGD, Pyro-DiRGD and Pyro-TriRGD in DMSO using DPBF as the scavenger. DPBF

concentration = $3 \times 10^{-5}$ M.

**5. The UV-Vis and Fluorescence spectra of Pyro-MonoRGD, Pyro-DiRGD, Pyro-TriRGD and free Pyro detected in DMSO.**

The Cary 5000 spectrophotometer(Varian Co., USA) was used for UV-Vis spectra. Absorption spectra were recorded from 300 to 800 nm. Fluorescence emission and excitation spectra were recorded from 550 to 800 nm upon excitation at approximately 668 nm and emission at 672-673 nm using a Hitachi Model F-4500 FL spectrophotometer (Tokyo, Japan).

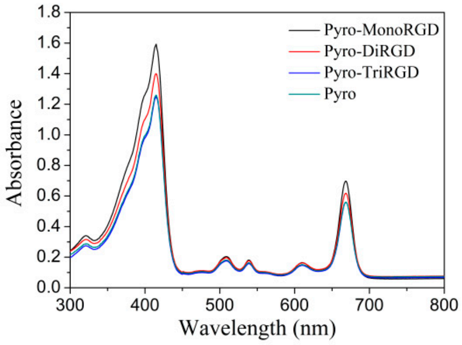


Fig. S23. UV-vis absorption spectra of Pyro-MonoRGD, Pyro-DiRGD, Pyro-TriRGD and Pyro in DMSO. The concentration for the absorption spectrum determination was 10 μM.

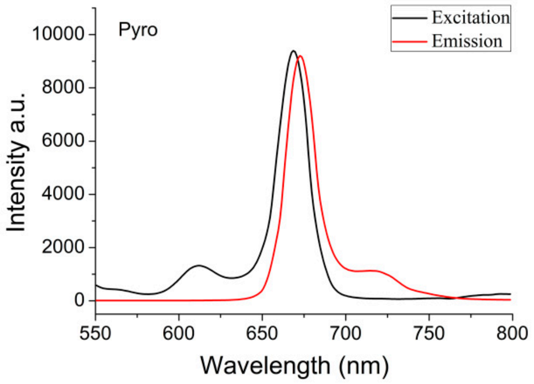


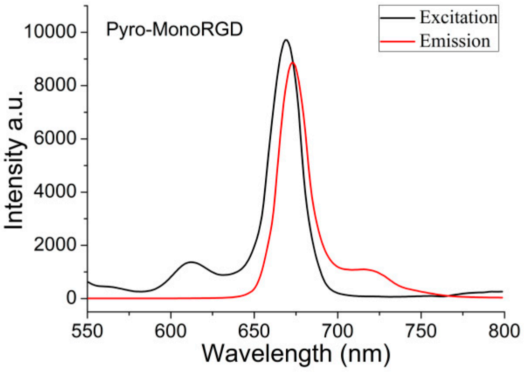

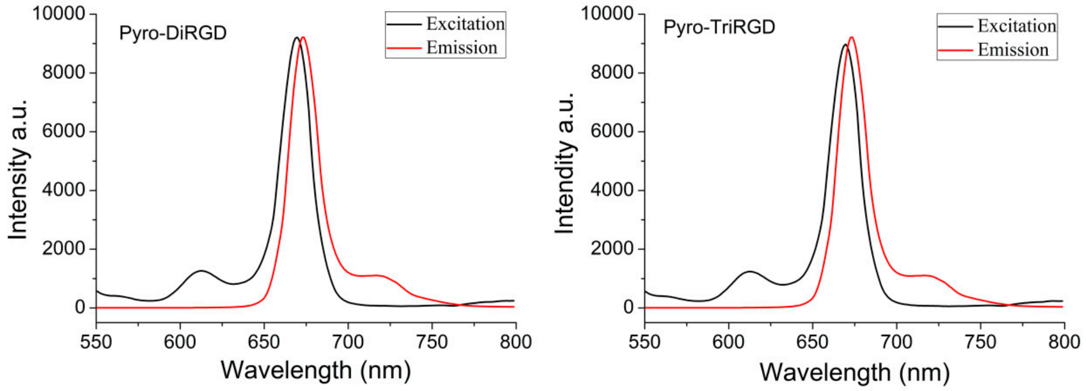


Fig. S24. Fluorescence absorption spectra of Pyro-MonoRGD, Pyro-DiRGD, Pyro-TriRGD and Pyro in DMSO. The concentration for the fluorescence determination was 2.0 μM.
